# An unconstrained palmprint region of interest extraction method based on lightweight networks

**DOI:** 10.1371/journal.pone.0307822

**Published:** 2024-08-09

**Authors:** Chengrui Lin, Yifan Chen, Xiangqun Zou, Xiaoling Deng, Fen Dai, Junchao You, Jinggui Xiao

**Affiliations:** 1 College of Electronic Engineering (College of Artificial Intelligence), South China Agricultural University, Guangzhou, China; 2 Guangzhou Intelligence Oriented Technology Co., Ltd., Guangzhou, China; 3 Lingnan Modern Agriculture Guangdong Laboratory, Guangzhou, China; 4 National International Joint Research Center of Precision Agriculture Aviation Application Technology, Guangzhou, China; Vellore Institute of Technology: VIT University, INDIA

## Abstract

Accurately extracting the Region of Interest (ROI) of a palm print was crucial for subsequent palm print recognition. However, under unconstrained environmental conditions, the user’s palm posture and angle, as well as the background and lighting of the environment, were not controlled, making the extraction of the ROI of palm print a major challenge. In existing research methods, traditional ROI extraction methods relied on image segmentation and were difficult to apply to multiple datasets simultaneously under the aforementioned interference. However, deep learning-based methods typically did not consider the computational cost of the model and were difficult to apply to embedded devices. This article proposed a palm print ROI extraction method based on lightweight networks. Firstly, the YOLOv5-lite network was used to detect and preliminarily locate the palm, in order to eliminate most of the interference from complex backgrounds. Then, an improved UNet was used for keypoints detection. This network model reduced the number of parameters compared to the original UNet model, improved network performance, and accelerated network convergence. The output of this model combined Gaussian heatmap regression and direct regression and proposed a joint loss function based on JS loss and L2 loss for supervision. During the experiment, a mixed database consisting of 5 databases was used to meet the needs of practical applications. The results showed that the proposed method achieved an accuracy of 98.3% on the database, with an average detection time of only 28ms on the GPU, which was superior to other mainstream lightweight networks, and the model size was only 831k. In the open-set test, with a success rate of 93.4%, an average detection time of 5.95ms on the GPU, it was far ahead of the latest palm print ROI extraction algorithm and could be applied in practice.

## 1 Introduction

Biometric recognition technology utilizes universally existing and unique physiological features of individuals for identity recognition and verification. It offers advantages such as convenience, non-replicability, accuracy, and efficiency, and has been gradually applied in various aspects of people’s lives and work. Among the different biometric recognition technologies, fingerprint recognition, facial recognition, and iris recognition have become relatively mature and widely used in multiple fields. Jain et al. [1] evaluated several common biometric features and highlighted the advantages of palmprint recognition over other features:1.Compared to fingerprints, palmprints cover a larger area, providing more information and greater robustness. Palmprints are also more difficult to forge and offer higher security compared to fingerprints, which can easily leave traces on smooth surfaces.2.Unlike facial recognition, which is prone to instability due to factors such as plastic surgery, makeup, twins, and aging, palmprints have advantages such as resistance to changes and interference.3.Compared to iris recognition, palmprint recognition offers advantages such as lower equipment cost, lower environmental requirements, and user-friendliness. Furthermore, palmprint recognition has the potential for multimodal recognition by combining with other hand-related features such as fingerprints, palm vein patterns, and hand shape. This integration can achieve higher accuracy and stronger anti-interference capabilities in multimodal recognition. Therefore, palmprint recognition holds promise as a reliable and effective biometric recognition technology, offering various advantages and the potential for multimodal recognition.

Palmprint recognition has developed gradually from contact-based and constrained recognition to contactless and unconstrained recognition [2]. Compared to the former, the latter offers higher user-friendliness. On the one hand, it can avoid contact between users’ palms and devices, reducing the risk of disease transmission. On the other hand, it provides users with more freedom and convenience, enabling more convenient, quick, and efficient identity verification. However, unconstrained palmprint recognition in real-world scenarios also faces obvious challenges. In situations where hand postures, angles, backgrounds, and lighting conditions are uncontrolled, the extraction of the region of interest (ROI) from palmprints becomes significantly more difficult. Therefore, solving the problem of ROI extraction in unconstrained palmprint recognition has become one of the focal points of researchers in recent years.

**Fig 1:**
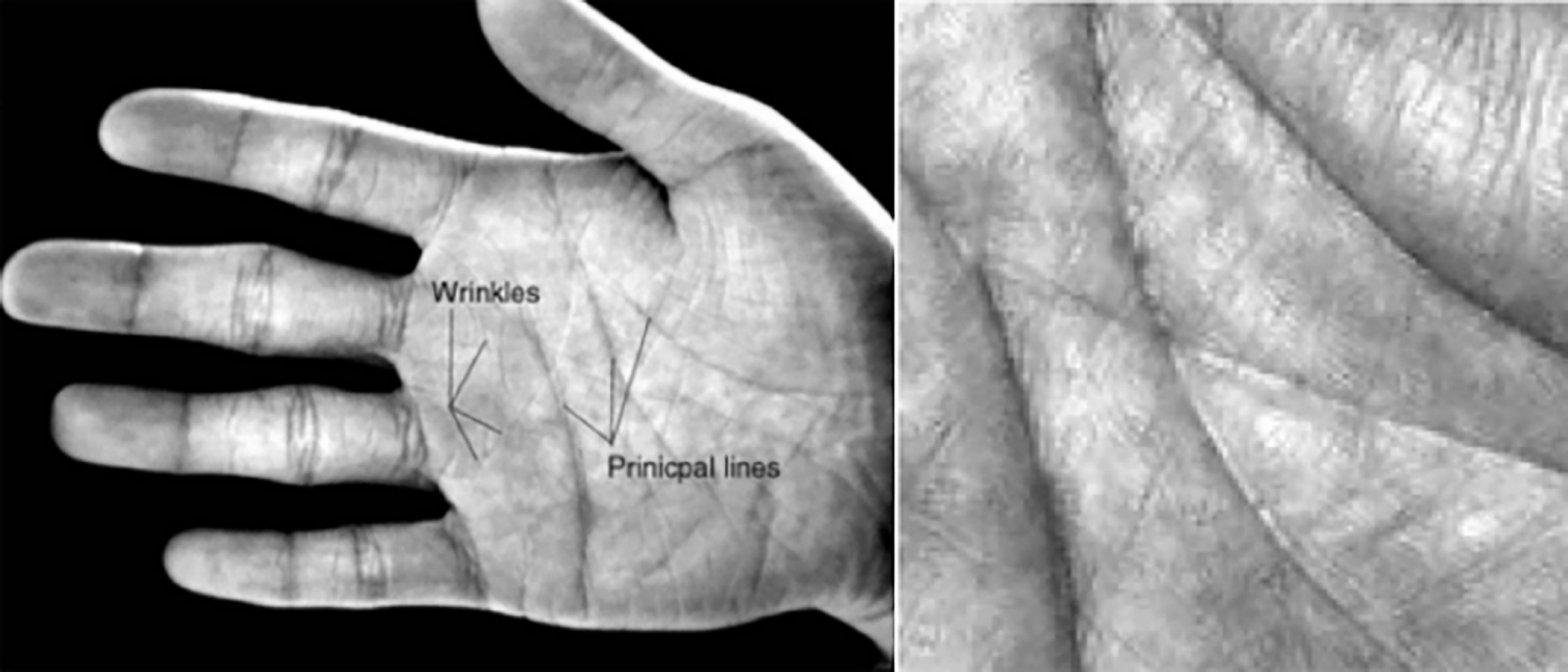
Main lines and fold characteristics of palmprint (left) and ROI (right).

**Fig 2:**
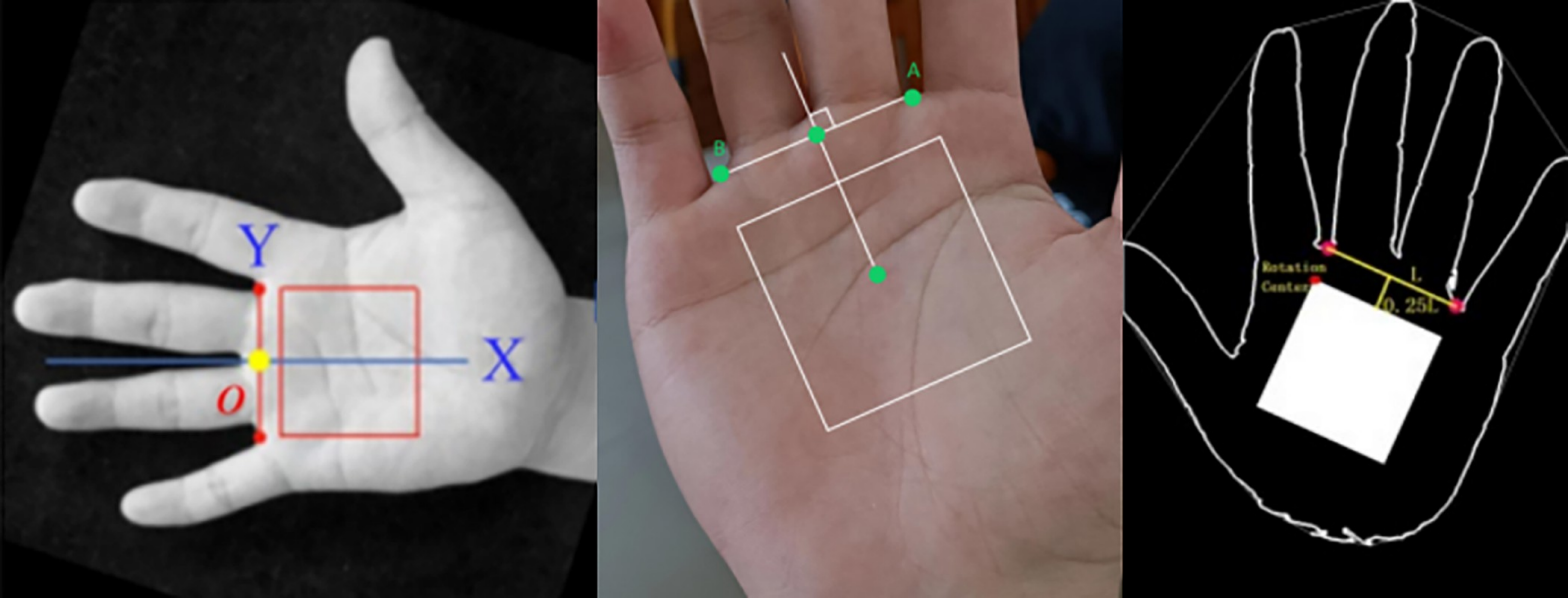
Illustration of the method of extracting ROI by keypoints positioning in literature.

The region of interest (ROI) in palmprint refers to the area in the center of the palm that contains three main lines and some creases, which hold rich information [3], as shown in Fig 1. There are two main methods for extracting the ROI: one is based on hand shape keypoints [4–9], and the other is based on inscribed circles [10–12]. The method based on inscribed circles is more time-consuming in searching for the inscribed circle and has poorer localization stability. On the other hand, the method based on hand shape keypoints can utilize these keypoints to perform orientation correction on the ROI and has faster localization speed, thus gradually becoming the mainstream research direction. The extraction process of the ROI based on hand shape keypoints, as shown in Fig 2, usually relies on valley points between the index and middle fingers and between the middle and ring fingers [13–15]. By establishing a coordinate system using these two valley points at a certain distance, the palm ROI can be located based on the corresponding distances within this coordinate system. Zhang et al. [3] proposed a line-based ROI extraction method. First, the palmprint image is subjected to low-pass filtering. Then, the hand region is separated from the background using the Otsu thresholding method to extract the palm contour. Subsequently, the edges of the index finger, middle finger, ring finger, and little finger are fitted using four straight lines. Finally, the valley points between the index and middle fingers and between the middle and ring fingers are used to locate the palm ROI region. Khan et al. [16] determine the starting point of the fingertips and palm by calculating the pixels in the hand region. After identifying the pixels corresponding to the valleys between the fingers, several second-order polynomials are used to extrapolate the midpoint of the valleys, and 70% of the palm width is determined as the area of the ROI. Zhou et al. [17] employed an ROI extraction method based on Zhang et al. [3] and Yoruk et al. [18]. They first determined the fingertip of the middle finger and extended it to 1.2 times the distance to the center of the palm. Using this point as a reference, the distance to all contour points was determined to detect the valleys and fingertips of the fingers. Kim et al. [19] utilized a hand contour detection method based on the YCbCr model. By analyzing the color information of the sample hand region pixels, they verified the presence of a hand in the image. Then, they detected the valley points of the fingers by sampling pixels from the determined hand region. Chai et al. [20] chose to use the R-grayscale component to remove the influence of lighting and proposed the PLS-FFCM method, which segments the palm through fast C-means clustering, and finally uses the convex hull convex depression algorithm to locate finger valleys.

With the development of deep learning, more and more researchers begin to use the method based on deep learning to extract palmprint regions of interest. Izadpanahkakhk [21] et al. proposed a fast CNN convolutional neural network (REM) inspired by AlexNet to detect the target in the palmprint region without keypoints location and palm segmentation, and extract ROI directly. However, the experiment was only conducted under the database with pure black background and basically unchanged palm posture. Zhang [22] et al. proposed to detect the two "double finger gaps" between the middle finger and the ring finger and the central area of the palm through yolov3 tiny, establish a coordinate system according to the central point of the "double finger gap" and the central point of the palm area, and extract ROI. However, the angle and shape of the palm in its experimental database are not diversified enough. When the palm posture or tilt angle is relatively large, the positioning of keypoints according to the way of target detection may be biased, and the existence of the palm can be judged only when the "double finger gap" and the central area of the palm exist simultaneously. Ito [23] et al. proposed a palm segmentation network HandSegNet. By adding an encoder decoder structure similar to UNet, they improved the PSPNet with a pyramid pool structure. The network was used to segment the palm and the background, and then ROI was located through valley points detection. In fact, this method improves the traditional method by using deep learning, but it is tedious to use segmentation method to detect valley points, and does not give full play to the advantages of deep learning. Luo [24] and Shao [25] first used yolov3-tiny to detect the palm and remove the irrelevant background, then used the network with the backbone network of Mobilenetv2 to detect 14 keypoints of the palm, and finally used the keypoints of the three valley points of index finger middle finger, middle finger ring finger and ring finger little thumb to establish a coordinate system and extract ROI. At the same time, an auxiliary network is introduced to estimate the palm angle during training to improve the convergence speed and accuracy of the model. This method draws on the idea of target detection in the first step of face recognition, which is worthy of reference, but its subsequent keypoints detection process is too cumbersome, and does not consider the size and running time of the two models in practical application. X. Liang [26] proposed a two-stage network PKLNet for hand keypoint localization. In the first stage, the palm image is segmented, the palm contour is extracted, and then the contour is fed into the keypoints detection network to detect valleys and ROI corners, and then the ROI is located. Although this method has high positioning accuracy and strong robustness, the model size is too large and the implementation is relatively cumbersome. Therefore, it can be said that there is currently no research focused on using lightweight deep learning methods to extract palm print ROI.

At present, the extraction of palm print ROI is often used as a preprocessing operation in palm print recognition systems, by extracting palm print ROI from the dataset for feature extraction and matching research. However, practical application scenarios are not like single, manually constructed datasets, often presenting more unpredictable challenges. In addition, it is precisely because previous studies only treated palm print ROI extraction as a preprocessing operation for palm print recognition that experiments are often run on PCs. The powerful processors on PCs often do not require researchers to consider the size and speed of the model, which is currently a gap in palm print ROI extraction research.

Therefore, this paper draws on the idea of target detection in the first step of face recognition [27–30], and introduces the idea of thermograph regression of face and human keypoints, and proposes a two-stage palmprint ROI extraction method based on depth learning. In addition, considering the feasibility of deploying to embedded devices, the lightweight network structure is adopted for the two stage network models, to fill the current research gap on lightweight network extraction of palm print ROI. The following are the main contributions of this paper:

1. An unconstrained palmprint database consisting of 178 hands of 89 people has been established, including how many pictures. The database contains different postures and angles (such as opening, closing, bending, tilting, etc.) and different lighting environments (such as normal lighting and the use of flashing lights) to simulate various situations that may occur in practical applications.
2. In the first stage of palmprint ROI positioning, the YOLOv5-lite lightweight network is used to initially locate the palm position, detect and extract the palm. In the second stage, an improved lightweight UNet network is used to locate the keypoints of the index finger middle finger valley point and the ring finger little thumb valley point of the palm ROI, and then the coordinate system is established according to the two keypoints, Extract the final palm ROI.

Contribution 1 can increase the diversity of samples for ROI research in palm print recognition, enhancing the robustness and generalization ability of algorithm research. Contribution 2 fills the current gap in this research field by proposing a method for extracting palm print ROI that takes into account model size, speed, and accuracy, and has good robustness and generalization ability, which can meet the needs of practical applications.

The main content of this paper is divided into four parts. The second part introduces the proposed method, the third part verifies the feasibility of the proposed method through experiments on multiple databases, and the fourth part is the conclusion.

## 2 Methods

Ethics Statement: All participants have agreed to have their images published together with the manuscript, as these images will not disclose any other information about the participants.

### 2.1 Process structure

**Fig 3:**
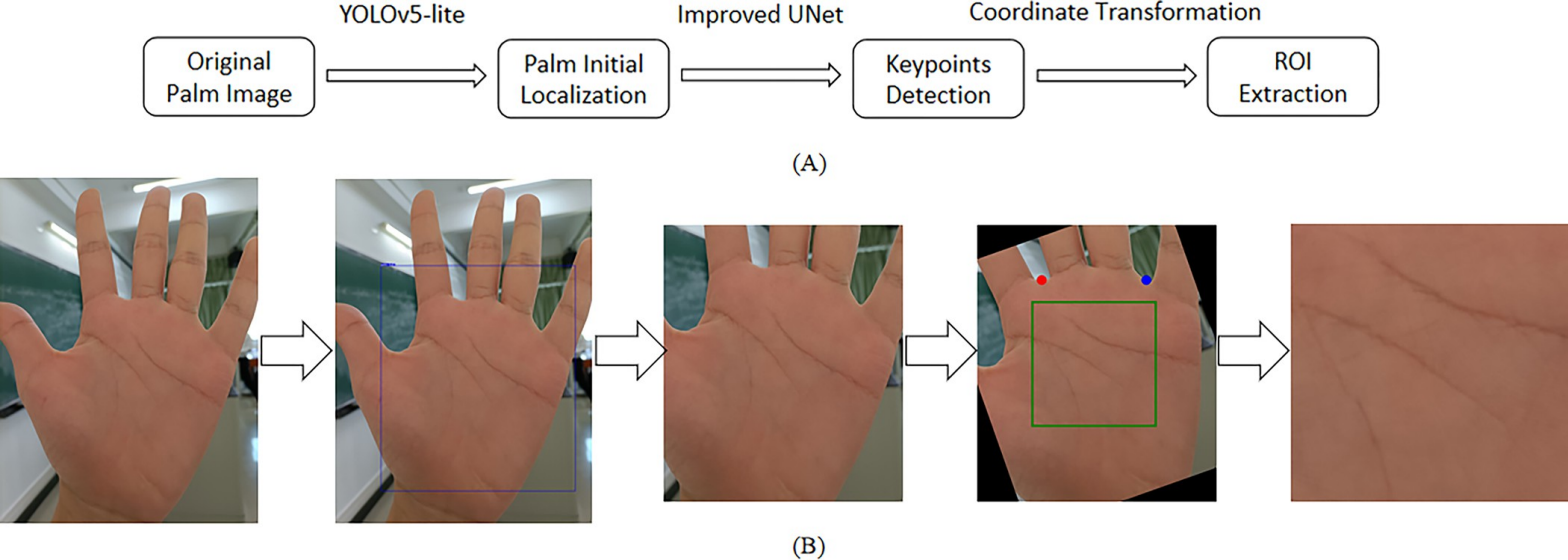
Flow structure of the proposed method.

As shown in Fig 3(A) and 3(B). Input them into the keypoints positioning network based on improved UNet, locate the keypoints of index finger middle finger valley and ring finger little thumb valley, and record them as point A and point B respectively (red and blue points in Fig 3(B). Calculate the included angle between the line between point A and point B and the horizontal line θ, And rotate the corresponding θ To achieve direction correction. Finally, a rectangular coordinate system is established with the two points connecting as the x axis and the center perpendicular of the two points connecting as the y axis. The point 0.8 | AB | away from the origin and located on the y axis is set as the ROI center, and the square area with 1.2 | AB | as the side length in the center is set as the ROI area and intercepted.

### 2.2 Datasets

**Fig 4:**
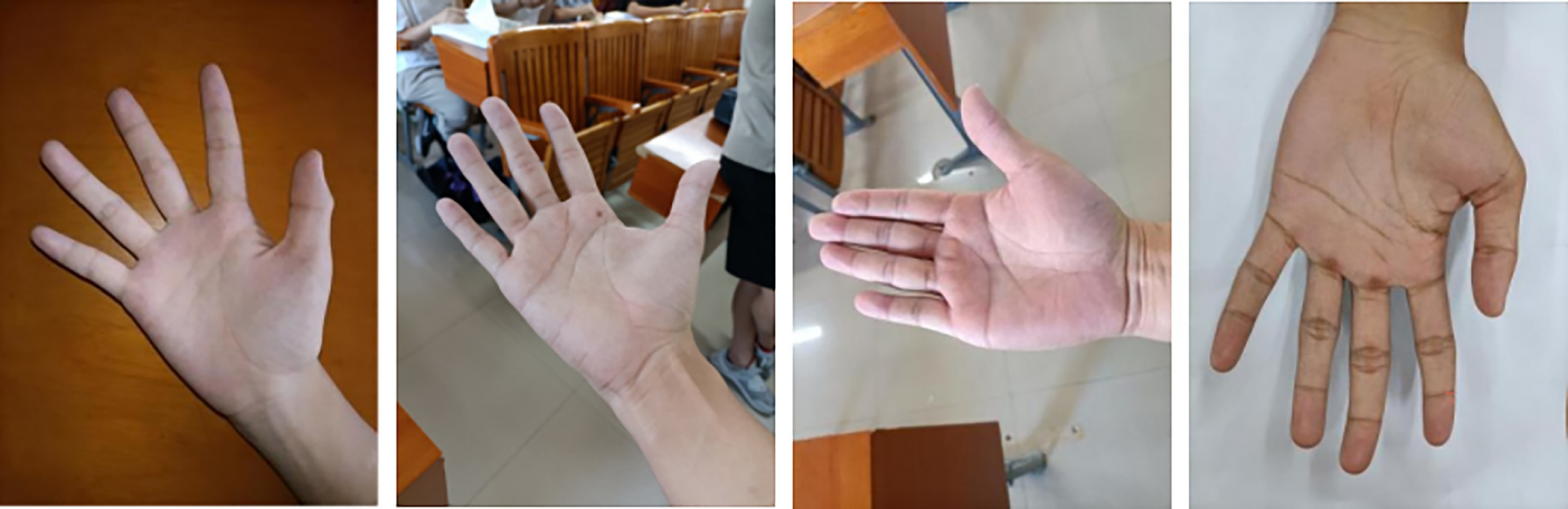
Example images of SCAUPD.

In order to better deal with the problems caused by various complex backgrounds, lighting, and hand postures and angles, and meet the needs of practical applications, South China Agricultural University has established a palmprint database, named SCAUPD (South China Agricultural University Palmprint Database). From September to October 2022, 89 students’ palm images were collected, each including left and right hand images, and each hand contains 10–20 images, including different lighting conditions—natural light conditions and flashing light conditions. There are 2274 pictures of different backgrounds—multiple occasions and pure blank backgrounds, different postures and angles of hands—vertical, horizontal, inclined, open, closed, curved, etc. The pictures are taken by smart phones, the size is 3000×4000, and the corresponding personnel information is marked. Some example images of SCAUPD are shown in Fig 4.

**Fig 5:**
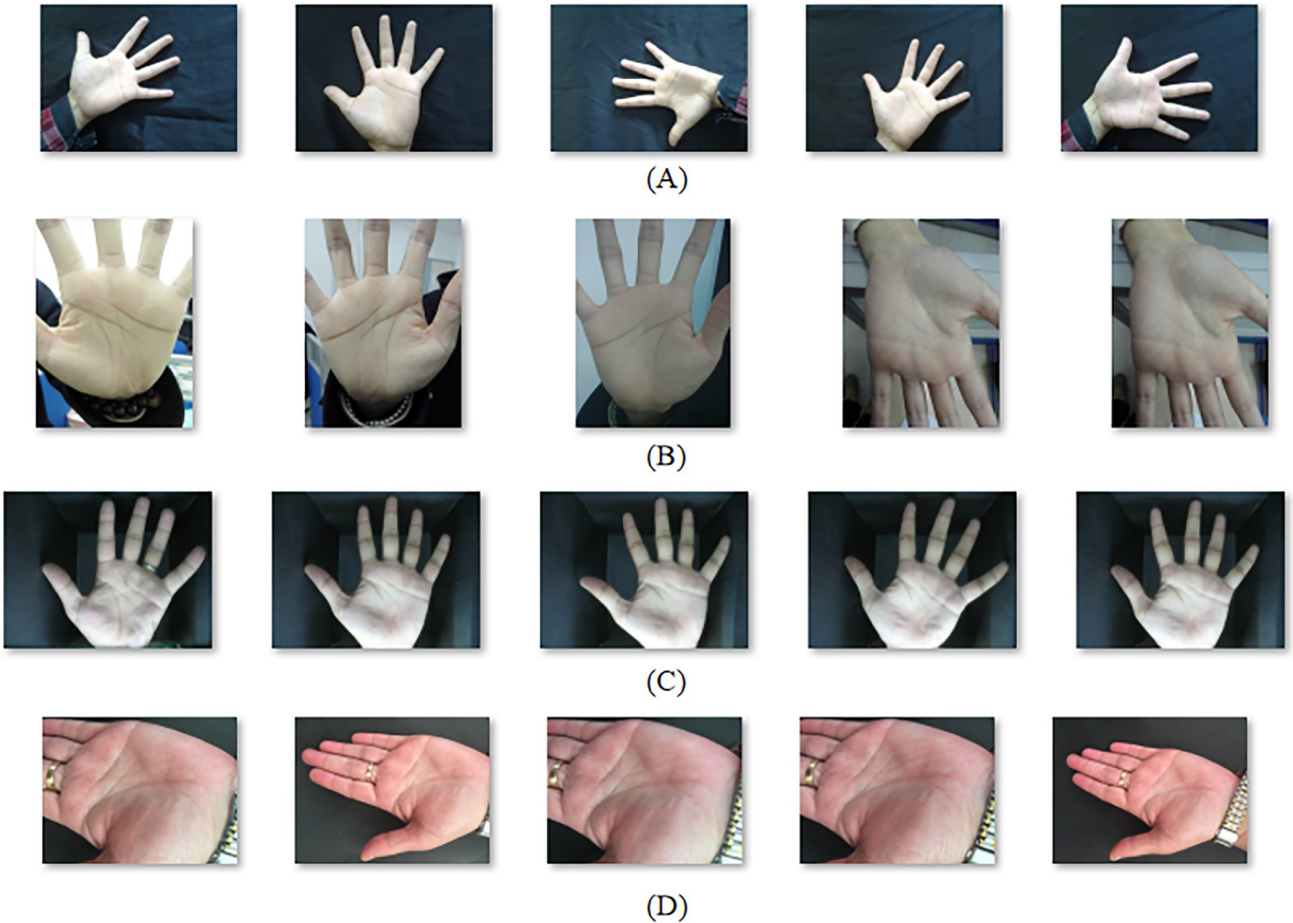
Example images of the database used: (A) REST; (B) MPD; (C) IITD; (D) BMPD.

In order to verify the performance of the proposed algorithm, BMPD (Birjand University Mobile Palmprint Database) [31], REST (REGIM Sfax Tunisian hand database) [32], MPD (Tongji Mobile Palmprint Database) [22] and IITD (Delhi Touchless Palmprint Database version 1.0) [33] were also used in the experiment. BMPD collected the left and right hand photos of 41 Iranian women, which were taken in two stages, with an interval of two weeks. There were 20 photos in each stage, and a total of 40 photos for one person. The BMPD did not control the light source, background, angle, posture and movement of the hands, and the distance between the hands and the lens. Moreover, because it was a female database, the proportion of wearing accessories on the hands was high. MPD collected photos of the left and right hands of 200 people in Tongji University, which were taken by two sessions and two smart phone devices. In one session, 10 photos were taken by each hand and each device. The REST database collected the left and right hand photos of 150 people aged 6–70. Although the background is pure and black, it does not control the posture and angle of the hands. IITD was taken from 2006 to 2007, and officially released in 2008. It collected the left and right hand photos of 230 people aged 14–56, which were taken by CMOS cameras. It is one of the earliest non-contact palmprint databases. Fig 5 is an example picture of the database used.

The reason for choosing the above 5 databases is that, except for SCAUPD, the remaining 4 are all publicly available non-contact palm print datasets. Among them, except for IITD, the remaining databases are all taken in close proximity to the present time, mostly using mobile phones or CMOS cameras, and there are no restrictions on hand posture and placement, which are more closely related to practical life applications. The previous ROI extraction methods often only targeted a specific dataset, or for situations where the background is relatively pure and the hand posture is relatively single, which is unfavorable for achieving application implementation.

### 2.3 Palm initial localization

In practical applications, the palmprint recognition system needs to first judge whether the target is a hand, and then can carry out the following ROI location, feature extraction and matching work. If the next operation is carried out directly without first judging whether the target is a hand, when multiple objects pass by or enter the camera shooting range at the same time, it will increase a lot of interference and affect the work of the recognition system. At the same time, after the initial localization of the palm, it can effectively extract the palm part, filter out most of the interference from the complex background, which is more conducive to the positioning of keypoints and ROI in the second stage. Since the main focus of palmprint recognition system is ROI location, feature extraction and matching, the applied network only plays an auxiliary role in the first stage of initial palm location, and lightweight, fast and efficient target detection algorithm should be considered. YOLO (You only look once) [34] redefines target detection as a regression problem. It applies a single convolutional neural network (CNN) to the entire image, divides the image into grids, and predicts the class probability and boundary box of each grid. Its detection speed is much faster than the two-stage detection algorithms R-CNN [35], Fast R-CNN [36], and Faster R-CNN [37]. It is one of the most powerful target detection algorithms today. YOLO algorithm has developed from YOLOv1 to YOLOv8, and each version has its own improvements compared with the previous version. Among them, YOLOv5-lite [38] is a lightweight version of YOLOv5 [39], which can be used to Map@0.5 When it reaches 35.1%, only 0.73G Flops and 0.78M parameters are used, and the scale is only 1.6M, which is very easy to deploy on various low computing power devices.

**Fig 6:**
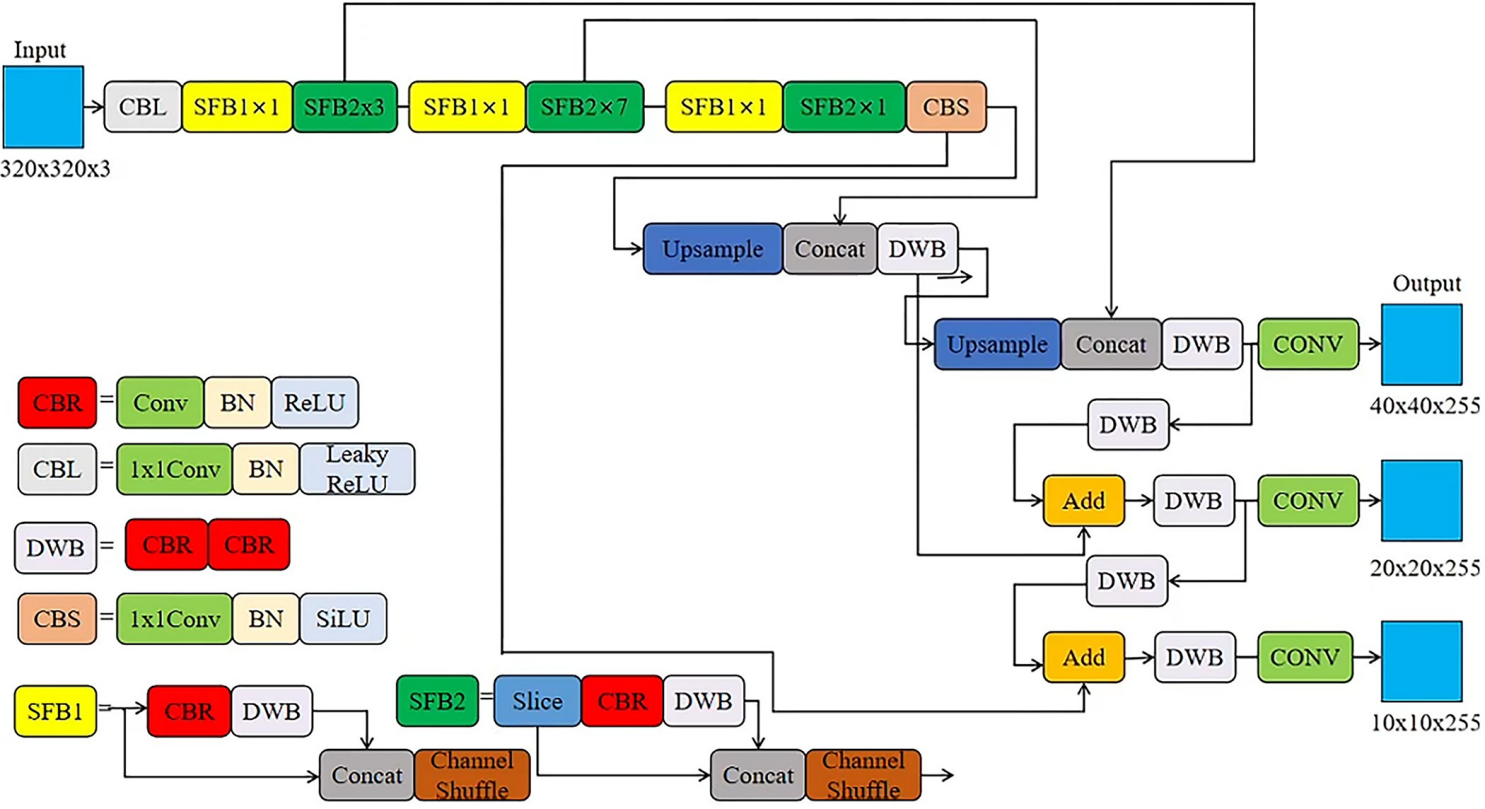
YOLOv5-lite network structure.

YOLOv5 lite, based on the lightweight design concept of shufflenetv2, replaced most of the convolution blocks of the backbone network with shufflenetv2 convolution blocks containing shuffle channels, reducing the amount of computation and parameters; 1024 conv and 5 of shufflenetv2 backbone are removed×5 pooling, greatly improving the detection speed without affecting too much accuracy; The Focus layer is removed to avoid excessive cache usage due to frequent slice operations; The C3 Layer with a high number of channels used in YOLOv5 is discarded to reduce the cache space and improve the running speed. In addition, YOLOv5-lite also prunes the head of YOLOv5 to minimize memory access. The network model of YOLOv5-lite is shown in Fig 6.

**Fig 7:**
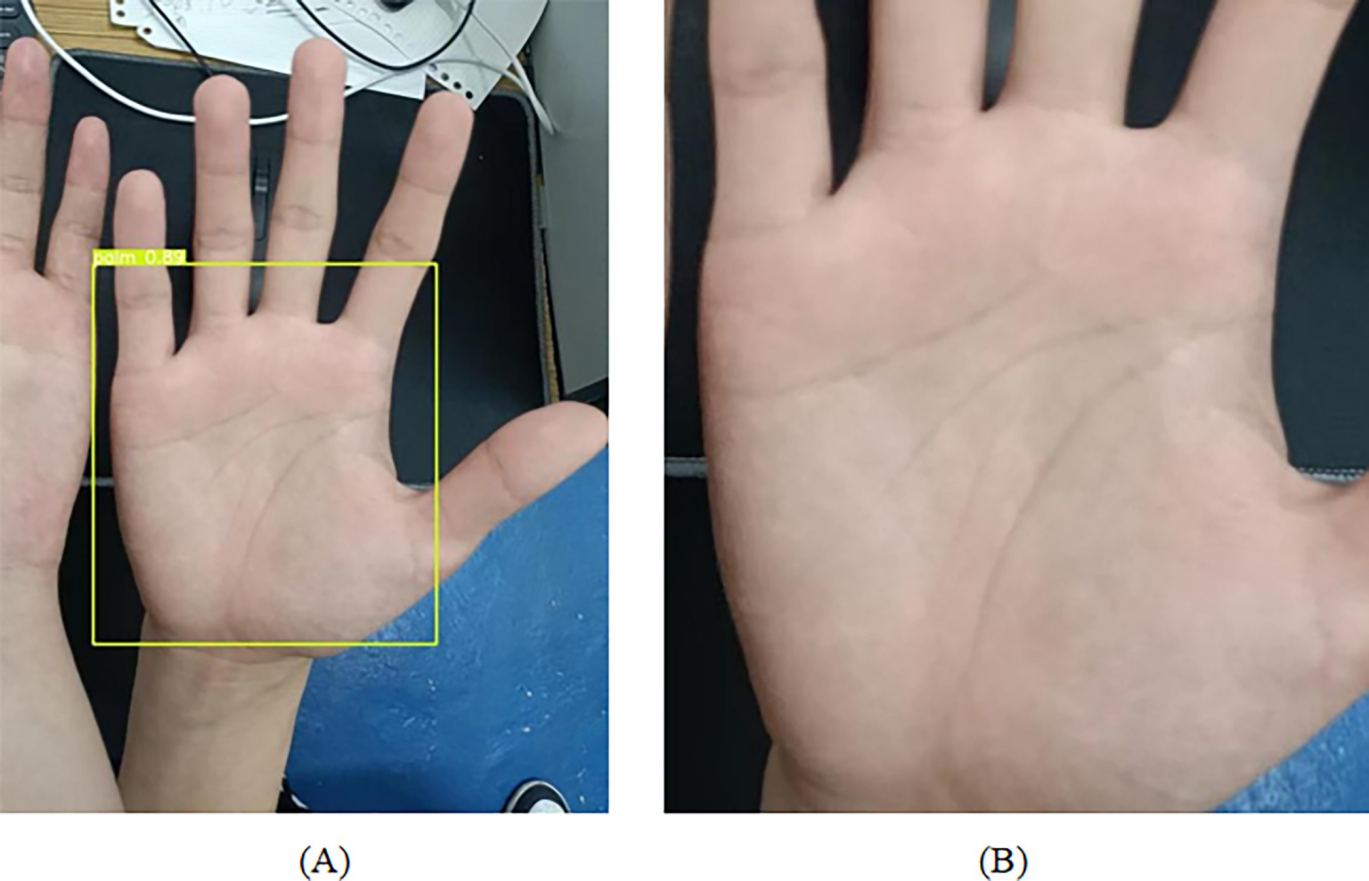
YOLOv5-lite palm initial localization output.

Fig 7 shows the output of the YOLOv5-lite network applied to palm initial localization. The model detected the palm as shown in Fig 7 (A), and captured the coordinates of the corresponding boxes to obtain Fig 7 (B). When multiple hands or subjects appear simultaneously in real-world application scenarios, it is difficult to accurately extract using a single palm print ROI localization network. On the one hand, interference increases due to factors such as hands or skin color, and on the other hand, the training set of the model often does not include such images because the training set is collected under specific circumstances, which cannot be avoided in practical applications. Therefore, achieving initial localization of the palm through YOLOv5-lite can effectively solve the above problems, transforming originally unpredictable, complex, and variable features into easily recognizable, simple, and obvious features for subsequent keypoints localization.

### 2.4 Keypoints positioning

**Fig 8:**
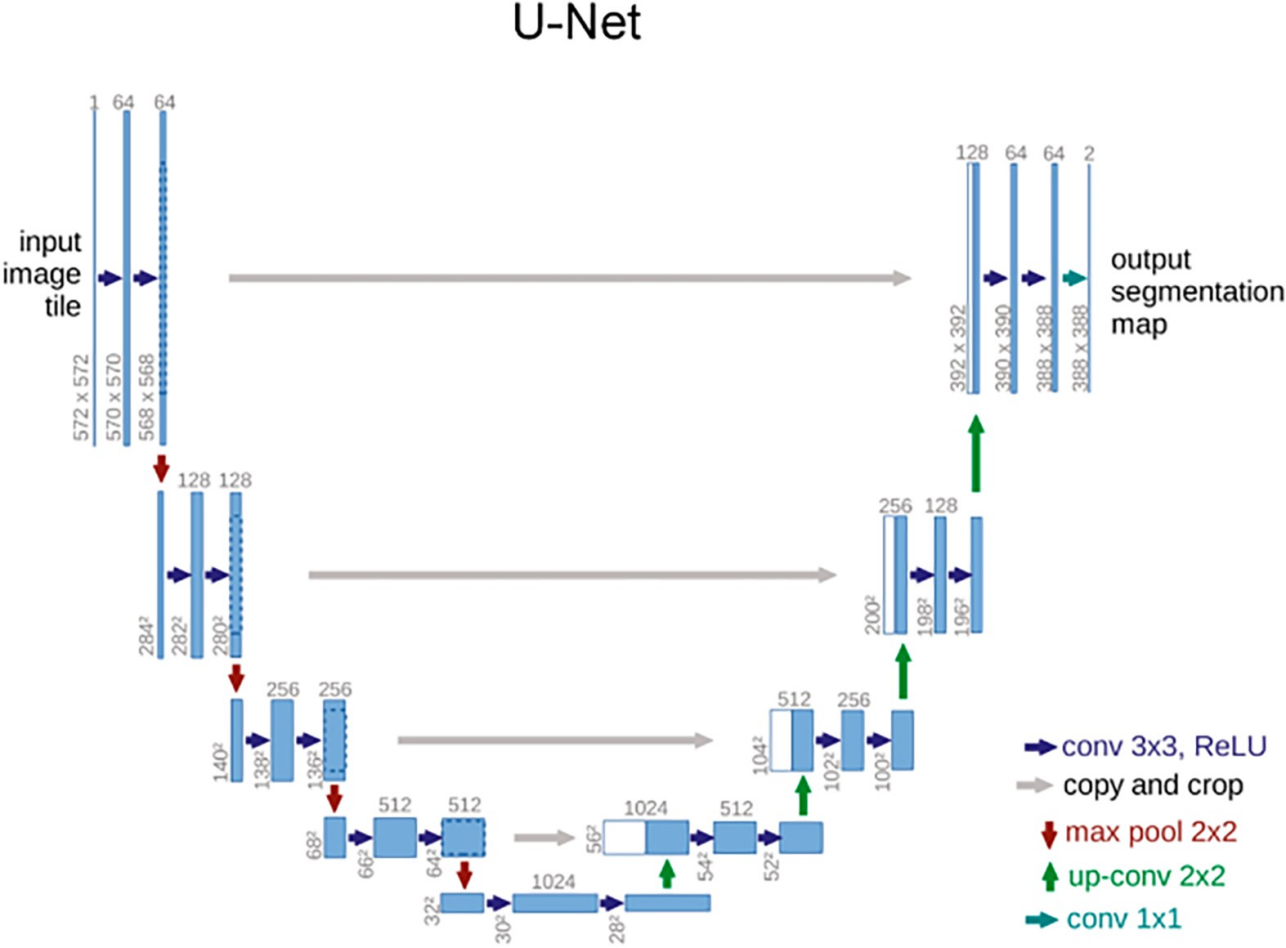
UNet network structure.

The palm image captured through YOLOv5-lite initial localization has obvious features: palm covering most of the area, four slender knuckles of fingers, and 3~4 obvious valleys between fingers. The traditional method of finger valley points detection is to separate the palm from the background, detect the palm contour, and then detect the valley points of the contour. Therefore, the valley points detection can be regarded as a segmentation problem. In the field of deep learning image segmentation, U-Net [40] has attracted wide attention. The innovation lies in the adoption of the encoder decoder structure, and the connection of the characteristics of the encoder and decoder through the jump connection. This design not only increases the perception ability of the network, but also can capture the features of multiple scales. At the same time, it combines the local information of high resolution and the overall information of low resolution, thus improving the accuracy of the segmentation results. The original U-Net network structure is shown in Fig 8.

However, directly defining keypoints localization as a segmentation problem not only increases the difficulty of image annotation and adds a lot of tedious work, but also fails to fully leverage the advantages of deep learning, which is too "non intelligent". In addition, segmentation networks often require larger scale models, rely more on powerful processors, and often need to run on PCs. However, actual embedded devices cannot have processors and memory comparable to PCs, so a more lightweight model and faster ROI extraction method are needed. Therefore, this article adopts a method of detecting keypoints rather than segmentation to achieve ROI extraction, as points detection is often much faster than edge segmentation. The model was constructed using the encoder decoder concept of U-Net, as points detection ultimately requires returning to the original image. Therefore, a network structure that downsamples through the encoder and upsamples through the decoder to the size of the original image is much more efficient, as will be demonstrated in subsequent experiments. Unlike other keypoints detection methods used for ROI localization, previous research on ROI keypoints detection has either detected four valleys between fingers, or detected more than ten keypoints located at the edge of the palm that surround it. The above process is too cumbersome. The keypoints detection network used in this article only detects two valleys directly related to ROI extraction, removes other inefficient steps, improves the speed of the network, and achieves lightweight, fast, and efficient keypoints detection.

**Fig 9:**
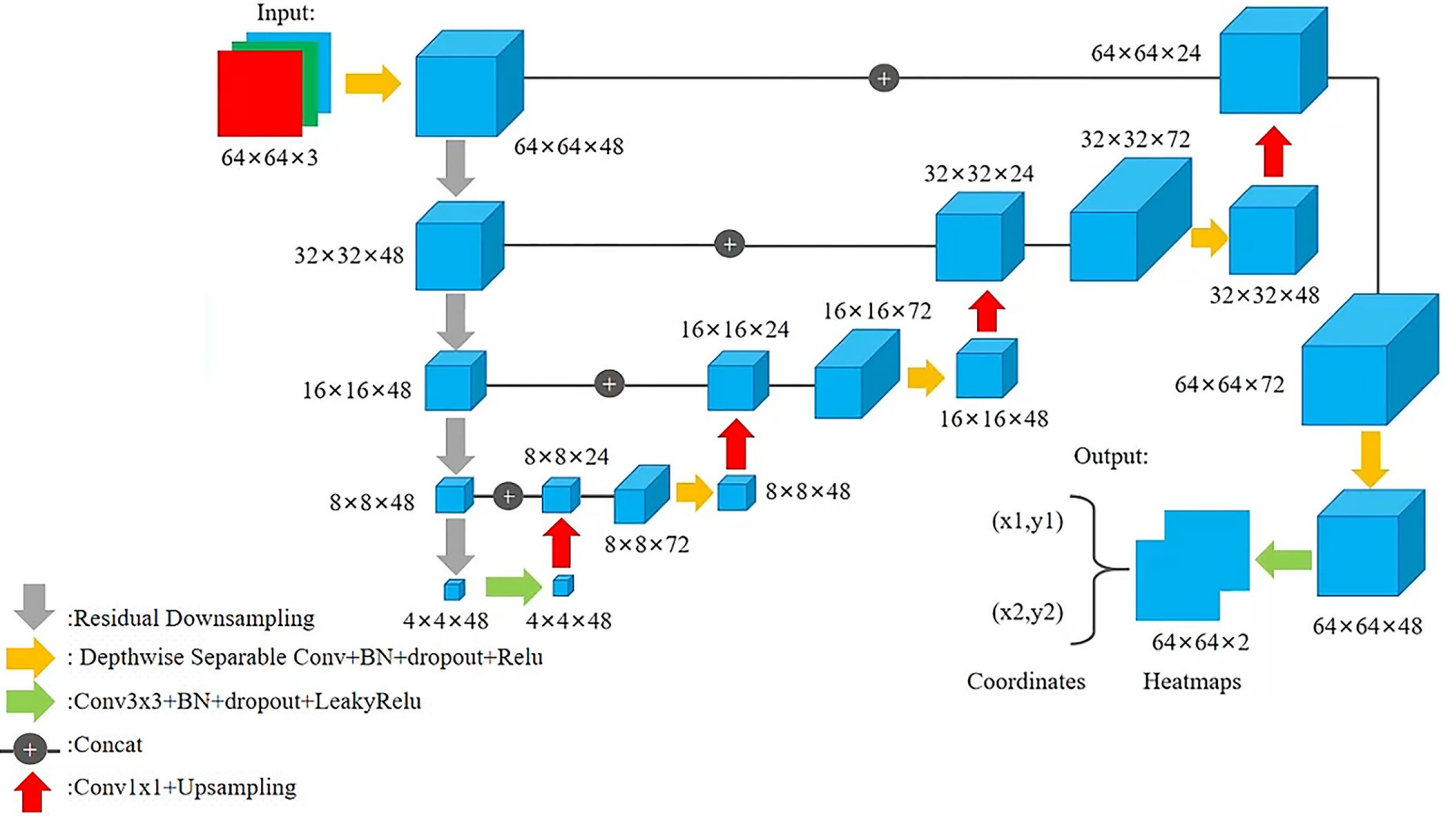
Proposed network structure.

The proposed network, as shown in Fig 9, has made several improvements compared to the original UNet in the following aspects:

1. Improved the original method of increasing the number of feature channels exponentially as the network depth deepens and the resolution of feature maps decreases. By reducing the number of convolutional kernels, the number of feature maps remains unchanged, avoiding the generation of too many redundant features and excessive computational complexity. The original UNet input image size is 572×572, and as the network deepens, the number of feature maps gradually doubles, from the initial 64 layers directly to 128 layers, 256 layers, and 512 layers. Finally, the bottom of the decoder reaches 1024 layers of feature maps, which undoubtedly generates excessive features and computational complexity. The reason why UNet is designed this way is because its original intention was to perform image segmentation. And our task is to only detect two key points, so the modification of UNet is necessary. Our network input has been changed to 64×64. A small-sized input means fewer convolution kernels and fewer parameters; At the same time, we only adopted the idea of UNet encoder decoder to downsample and upsample the input, abandoning the method of gradually doubling the number of original UNet feature maps. We fixed the number of layers of feature maps to 48, because the more feature maps there are, the more convolution kernels and convolution times are required, and the larger the data volume. Therefore, the improved network input image size is (64×64×3) After downsampling multiple times through the network, the bottommost point becomes (4×4×48), and finally upsampled to (64×64×2), where 2 represents a feature map with two keypoints.
2. The original UNet only used max pooling for downsampling, while we combined a stride of 2 convolution with residual connections to achieve downsampling. We call it "residual downsampling". Replacing the original method of downsampling through max pooling with using convolution with a stride of 2, and introducing residual connections to avoid network degradation caused by reduced feature count and network deepening. The gray arrows in Fig 9 represent residual downsampling.
3. By replacing the original ordinary convolution with separable convolution, the number of feature maps in each level of network is reduced from 48 to 72, an increase of 1/2. In this case, using ordinary convolution will greatly increase the number of parameters. Therefore, when the number of feature maps is 72, using depthwise separable convolution can maintain the same effect while reducing the number of parameters to one-third of the original, achieving lightweight network. The yellow arrows in Fig 9 represent depthwise separable convolution.
4. Modify the output layer of UNet. Due to its application in keypoint detection tasks rather than segmentation tasks, it is necessary to modify the output of the network. This article adopts a combination of Gaussian heatmap regression and direct regression, with the output end changed to two Gaussian heatmaps of the same size as the original image (64×64×2). The Gaussian heatmap represents the probability value of each pixel on the graph belonging to the label, and the pixel with the highest probability value is the coordinate point. Then, the soft argmax function is used to regress to the coordinates of the key point with the highest probability value in the corresponding heatmap. Therefore, the network has four outputs, namely two heat maps and coordinate arrays of two keypoints.

#### 2.4.1 Residual downsampling

**Fig 10:**
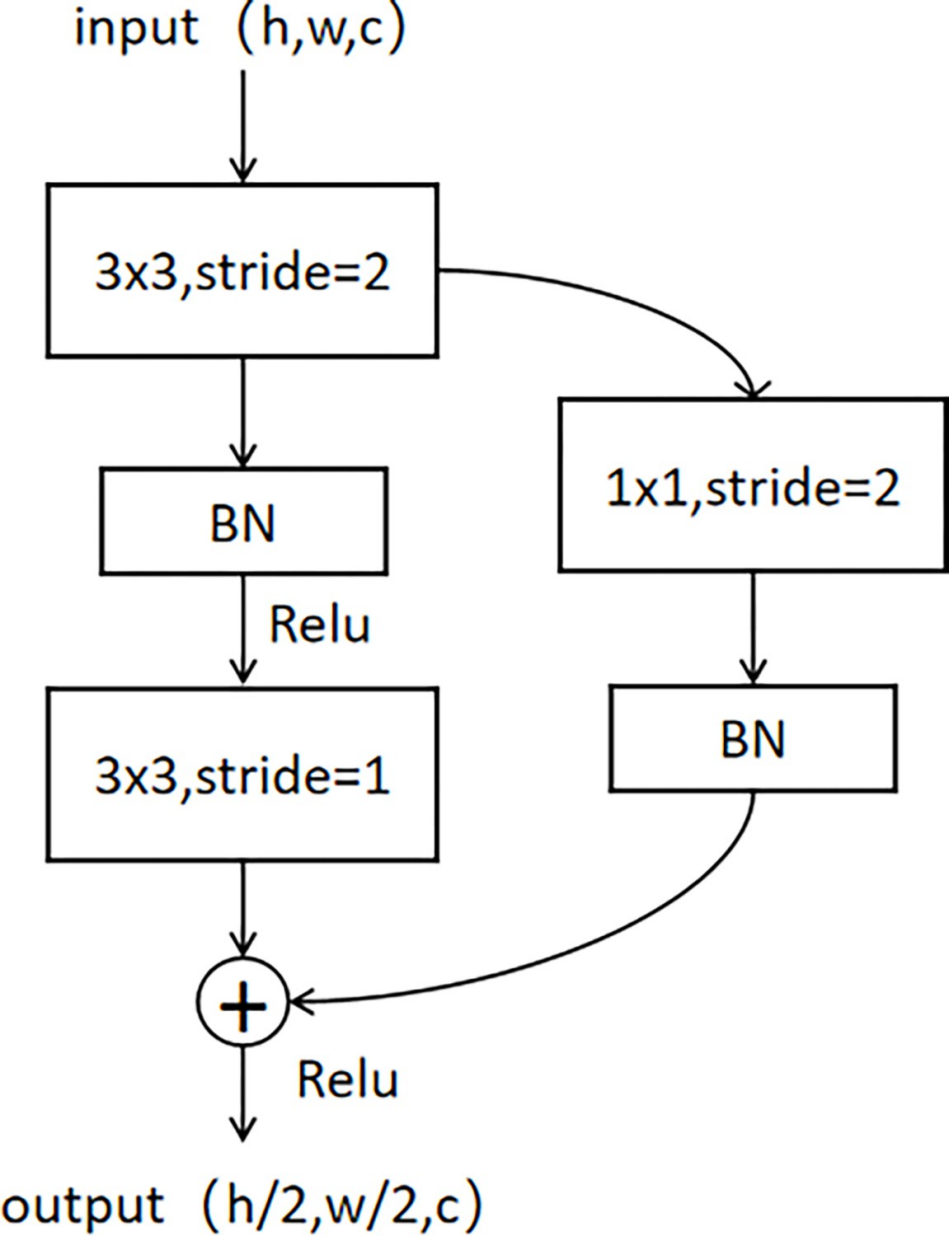
Example of residual downsampling, input size (h, w, c), output size (h/2, w/2, c).

The original U-Net used 2×2 max pooling to downsample the image, but max pooling directly calculates the raw data within the pooling window. Although it is faster, its downsampling process is not learnable and can easily filter out potentially useful information. The advantage of using convolution for downsampling is that it allows for feature extraction while downsampling, allowing the model to learn more information and accelerate model training. The residual downsampling used in this article is shown in Fig 10. The input image passes through two paths, one is a 3×3 convolution with a step size of 2, and the other is a 1×1 convolution with a step size of 2. This allows the model to learn richer features by passing through convolution kernels of different scales. Then, the feature map that has undergone 3×3 convolution is passed through the BN layer and Relu function, followed by a 3×3 convolution with a step size of 1, and then fused with the feature map that has undergone a 1×1 convolution path with a step size of 2. At this point, the output feature map becomes half of the original, while the number of channels remains unchanged.

#### 2.4.2 Depthwise separable convolution

**Fig 11:**
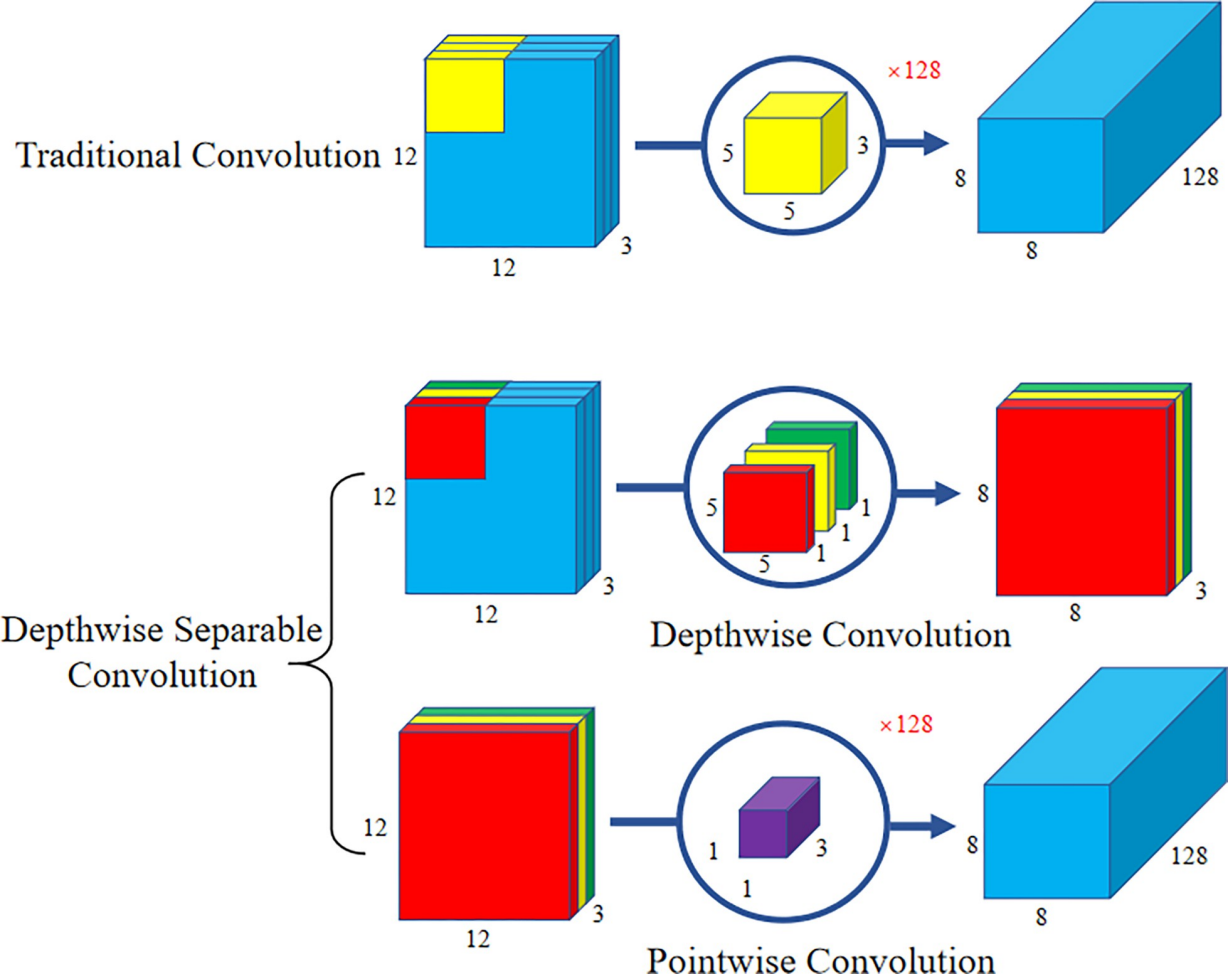
Depthwise separable convolution.

Traditional convolution operations involve point-wise cross-correlation calculations on input feature maps, where each output feature map channel is convolved with all input channels. This process results in a significant computational burden, especially when the number of channels is high. Therefore, the proposed network replaces standard convolutions with depthwise separable convolutions when convolving deep and high-channel feature maps to reduce the parameter volume of the network. Depthwise separable convolution is a decomposable convolutional structure that decomposes a standard convolution into depth-wise convolution and point-wise convolution. In this operation, each input feature channel is convolved separately using depth-wise convolution, and then the output of the depth-wise convolution is point-wise convolved using a 1x1 convolutional kernel. This decomposition process effectively reduces the number of parameters and computations in the model, speeding up the inference process. Furthermore, depthwise separable convolution also exhibits regularization effects. Due to the convolution operations performed on individual channels, it can limit the network’s representational capacity and encourage learning of more generalized features. Therefore, depthwise separable convolution performs well in preventing overfitting. The architecture of depthwise separable convolution is shown in Fig 11.

For an image with length *h*, width *w*, and number of channels ***c***_***in***_, using a traditional convolutional layer with a kernel size of (*k*, *k*) to convolve it, resulting in a cout output channel, the required convolution computation *n*_*conv*_ is:

$$n_{conv}=c_{in}\cdot h\cdot w\cdot k^{2}\cdot c_{out}$$ (1)

And depthwise separable convolution first performs depthwise convolution on the image, with a computational cost *n*_*depthwise*_ of:

$$n_{depthwise}=c_{in}\cdot h\cdot w\cdot k^{2}$$ (2)

Further perform point by point convolution on it, with a computational cost *n*_*point*_ of:

$$n_{point}=h\cdot w\cdot c_{out}$$ (3)

Therefore, the computational cost of depthwise separable convolution ***n***_***dsc***_ is the sum of depthwise convolution and pointwise convolution, which is:

$$n_{dsc}=n_{depthwise}+n_{point}=h\cdot w\cdot (c_{in}\cdot k^{2}+c_{out})$$ (4)

The ratio of the computational cost of depthwise separable convolution to that of traditional convolution is:

$$\frac{n_{dsc}}{n_{conv}}=\frac{1}{c_{out}}+\frac{1}{k^{2}}$$ (5)

It can be seen that as the number of output channels increases and the size of the convolution kernel increases, the ratio of computational complexity between depthwise separable convolution and traditional convolution decreases, which can effectively reduce the number of model parameters.

#### 2.4.3 Gaussian heatmap regression and direct coordinate regression

In face keypoints detection and body keypoints detection [41–45], Gaussian heatmap regression and coordinate regression are two different methods. The Gaussian heatmap regression method generates a heatmap of the same size as the original image to represent the location information of keypoints. For each target keypoint, a Gaussian distribution is generated on the heatmap, with its center corresponding to the keypoints’ position. The intensity of other pixels in the heatmap is determined based on their distance from the keypoints. Typically, the intensity decreases as the distance from the keypoints increases. When multiple keypoints are present, the final heatmap is obtained by overlaying individual keypoints heatmaps. The generation of Gaussian heatmaps is shown in (6).For label keypoint (*x*_0_, *y*_0_), given a Gaussian kernel *σ*, the generated Gaussian heatmap distribution is $p_{\left(x_{0},y_{0}\right)}$, where *x* and *y* represent the coordinates of each pixel in the image.

**Fig 12:**
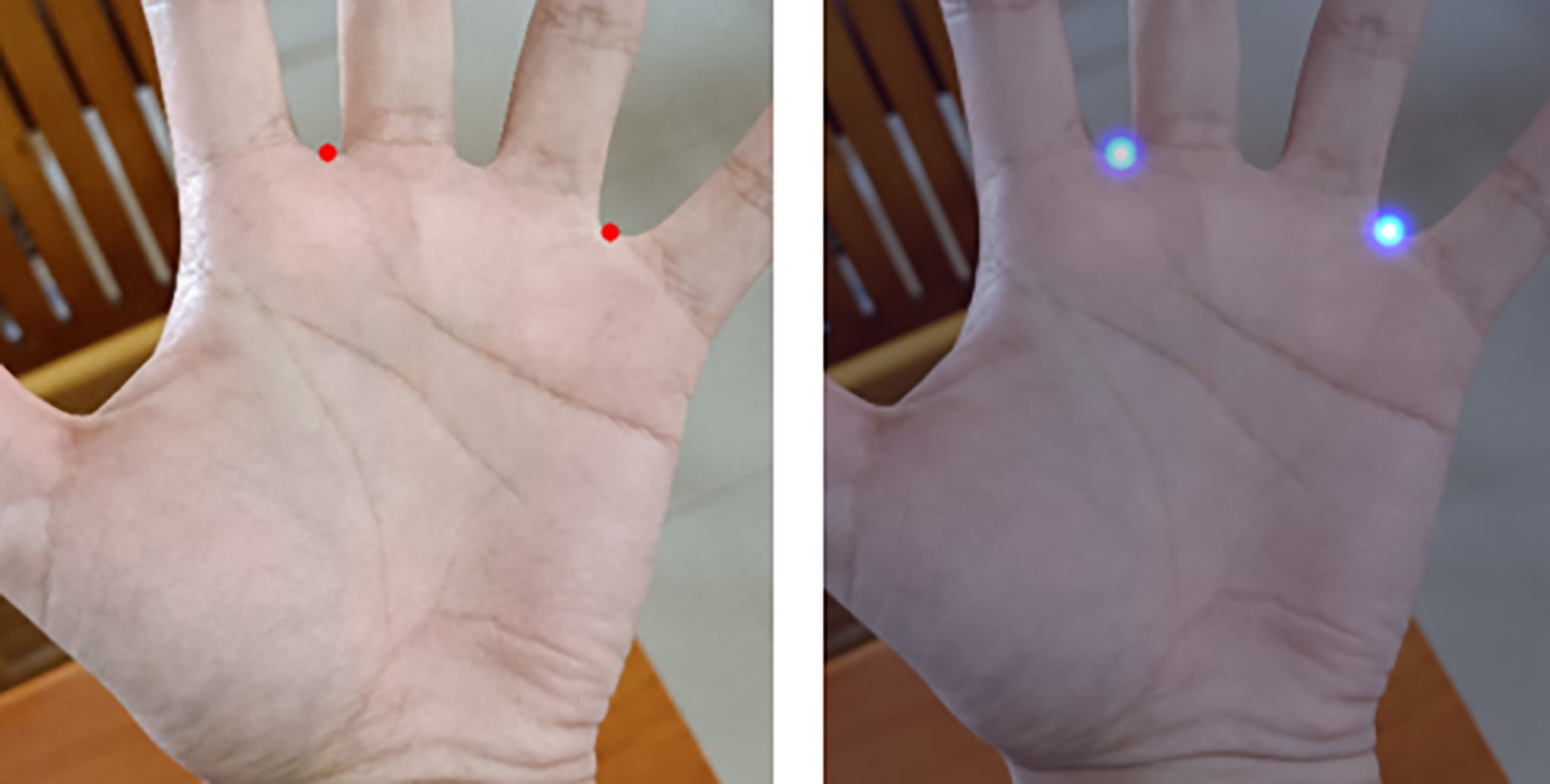
Keypoints in the original image (left) and keypoints visualized under the heatmap (right).

Compared to direct coordinate regression, Gaussian heatmap regression has higher robustness and generalization ability. It captures the spatial information of target keypoints and is more robust to image deformations, rotations, scale changes, and other variations, making it more suitable for application in various complex scenarios. Additionally, when detecting valley points, in large input images from smartphones with rich pixel and color information, valley points are not represented by a single pixel. The direct coordinate regression method can only select the coordinates of a single pixel as the label for supervision. This makes it challenging for the model to learn relevant features solely based on a single pixel. In direct coordinate regression, the model needs to convert spatial positions into coordinates during training, which ignores the spatial information of keypoints. The model can only optimize and learn through an extremely nonlinear process, leading to more difficulty in convergence. Moreover, the learned weights in training highly depend on the distribution of the training data, resulting in weaker robustness and generalization ability. On the other hand, the Gaussian heatmap regression method provides directional guidance to the network during training. The closer the distance to the target point, the larger the activation value, allowing the network to quickly reach the target point in a directed manner. Fig 12 shows the original annotated positions of keypoints and the visualization of the generated heatmaps.

Ethics Statement: All participants provided written informed consent and appropriate, photographic release. The individuals shown in Fig 12 have given written informed consent (as outlined in PLOS consent form) to publish their image.


$$p_{\left(x_{0},y_{0}\right)}=e^{-\frac{{\left(x-x_{0}\right)}^{2}+{\left(y-y_{0}\right)}^{2}}{2\sigma^{2}}}$$ (6)

However, compared to the Gaussian heatmap regression approach, direct coordinate regression directly predicts the coordinates of keypoints and can achieve higher precision in keypoints localization. Therefore, this paper combines Gaussian heatmap regression with direct coordinate regression. The soft-argmax function is used on the next layer generated by the heatmap to obtain the coordinates of the point with the highest probability in the Gaussian heatmap and incorporates it as another output of the network, which is then involved in the calculation of the loss function during training. The traditional argmax operation directly returns the integer index of the maximum value in the array. This method cannot calculate gradients during backpropagation because its output changes in a stepped form and there are no learnable gradients. The soft -argmax function is designed to overcome this difficulty. For a given set of scores, soft-argmax first converts each score into probability through the softmax function, and then calculates its expected value. This expected value can be seen as the weighted average index of this set of numbers, and the weight is the output of the softmax function. The softmax and soft-argmax functions are shown in (7) and (8), where *x* represents the score of the input, *j* represents the total number of indexes, and *i* represents the i-th index.


$$softmax(x_{i})=\frac{e^{x_{i}}}{\sum_{j}e^{x}}$$ (7)

$$soft-argmax(x)=\sum_{j}i*softmax(x_{i})$$ (8)

#### 2.4.4 Joint loss function

Since a combination of Gaussian heatmap regression and direct coordinate regression is used for keypoints detection, a joint loss function is proposed to guide the network learning. This new loss function combines the JS divergence loss between the computed heatmap prediction results and the labels, as well as the Euclidean loss between the coordinate prediction results and the labels. The JS divergence loss is based on the concept of Jensen-Shannon divergence, which measures the difference between two probability distributions. Its value ranges between 0 and 1, where smaller values indicate higher similarity between the distributions, while larger values indicate greater differences. JS divergence is a variation of KL divergence. For the i-th component of n input samples x, given two probability distributions P(x) and Q(x) representing the true distribution and the predicted distribution of the model, respectively, the KL divergence (Kullback-Leibler divergence) is defined as shown in (9).


$$KL(P||Q)=\sum_{i=1}^{n}P(x_{i})log\frac{P(x_{i})}{Q(x_{i})}$$ (9)

Since KL divergence is asymmetric, meaning KL(P || Q) ≠ KL(Q || P), the JS divergence was introduced to address this issue. The JS divergence is an improved version of KL divergence and is defined as shown in (10).


$$JS(P||Q)=\frac{1}{2}KL(P(x)||\frac{P(x)+Q(x)}{2})+\frac{1}{2}KL(Q(x)||\frac{P(x)+Q(x)}{2})$$ (10)

The L2 loss function is used to compute the distance error between the predicted coordinates of keypoints and their corresponding labels. For the i-th component y^i of n outputs of the model, with y_i_ denoting the true label for the corresponding component, we have (11).


L2(y^,y)=1n∑i=1n(y^i−yi)2 (11)

Lastly, by combining the JS divergence loss function and the L2 loss function, the final joint loss function of the model is defined as (12).


Loss=JS(Y^,Y)+L2(y^,y) (12)

Where Y^ and Y are the predicted and true distributions of the heatmaps, and y^ and y are the predicted and true coordinates of the keypoints, respectively.

### 2.5 ROI extraction

**Fig 13:**
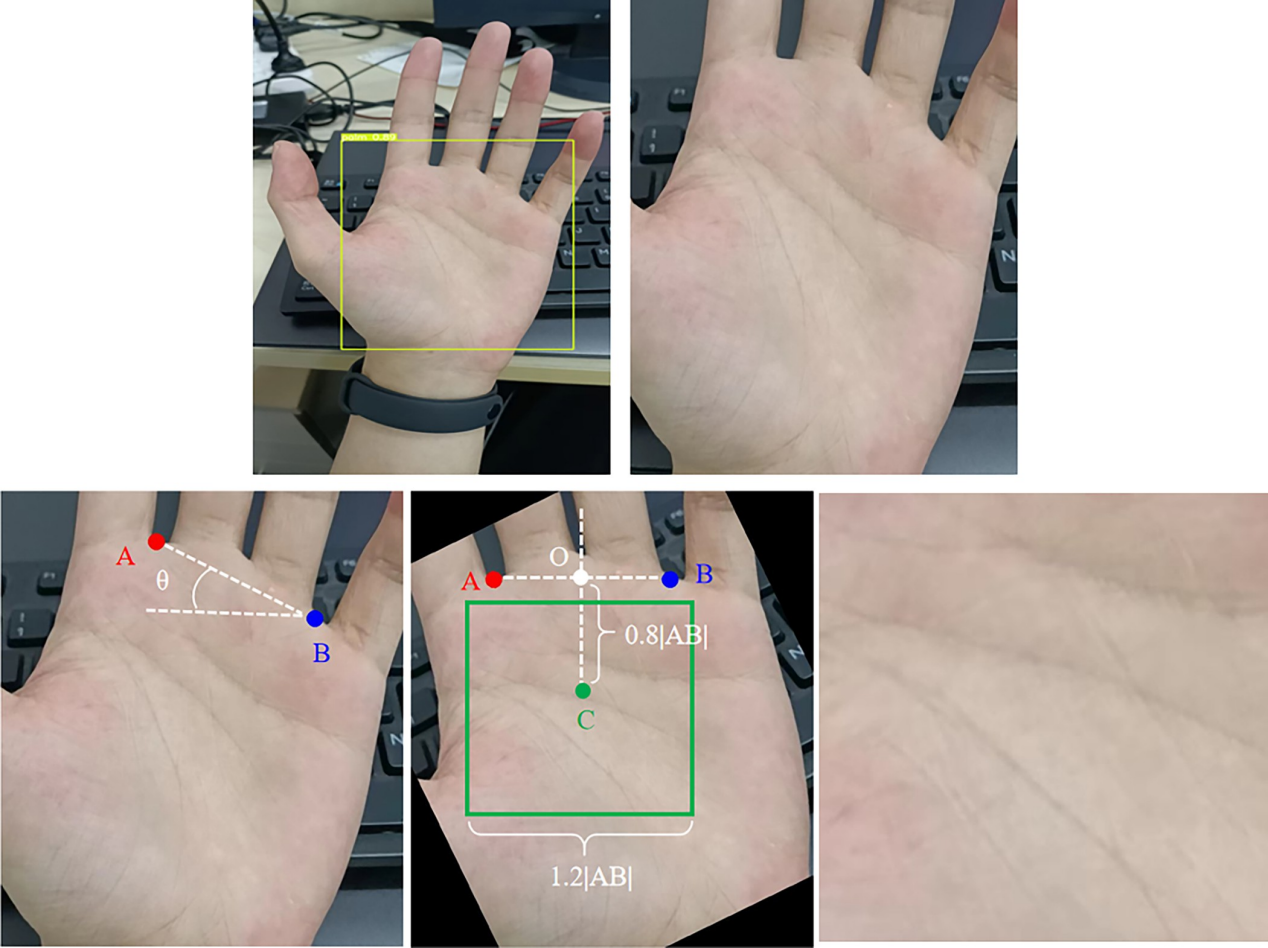
Schematic diagram of ROI extraction algorithm.

The extraction of ROI is shown in Algorithm 1, as illustrated in Fig 13.

Algorithm 1: ROI Extraction Process

Requirements: Hand image I, object detection network YOLO5-lite, and keypoints detection network Improved UNet

1:Input I to YOLO5-lite to obtain the palm localization image I1.

2:Input I1 to Improved UNet to detect two keypoints, A and B, where A represents the valley point between the index and middle fingers, and B represents the valley point between the ring finger and little finger.

3:Calculate the angle θ between the line AB and the horizontal direction, θ = arctan ($\frac{\left|yB-yA\right|}{\left|xB-xA\right|}$) and rotate the image by θ to correct its orientation.

4:Establish a Cartesian coordinate system with the line AB as the x-axis and the perpendicular bisector of AB as the y-axis, with the origin at O.

5:Set point C as the point on the y-axis, with a distance of 0.8|AB| from O.

6:Draw a square with point C as the center and a side length of 1.2|AB|, and extract it as the ROI of the palm print.

It should be noted that the selection of midpoint C and the setting of ROI edge length are flexible and variable, and may not necessarily be 0.8|AB| and 1.2|AB| in practical applications. This is also one of the advantages of this method, which is to determine the position of key points and flexibly set the ROI region to be intercepted based on different distance rules, enabling multi-scale ROI region extraction.

## 3 Experiment

### 3.1 Datasets and data preprocessing

**Fig 14:**
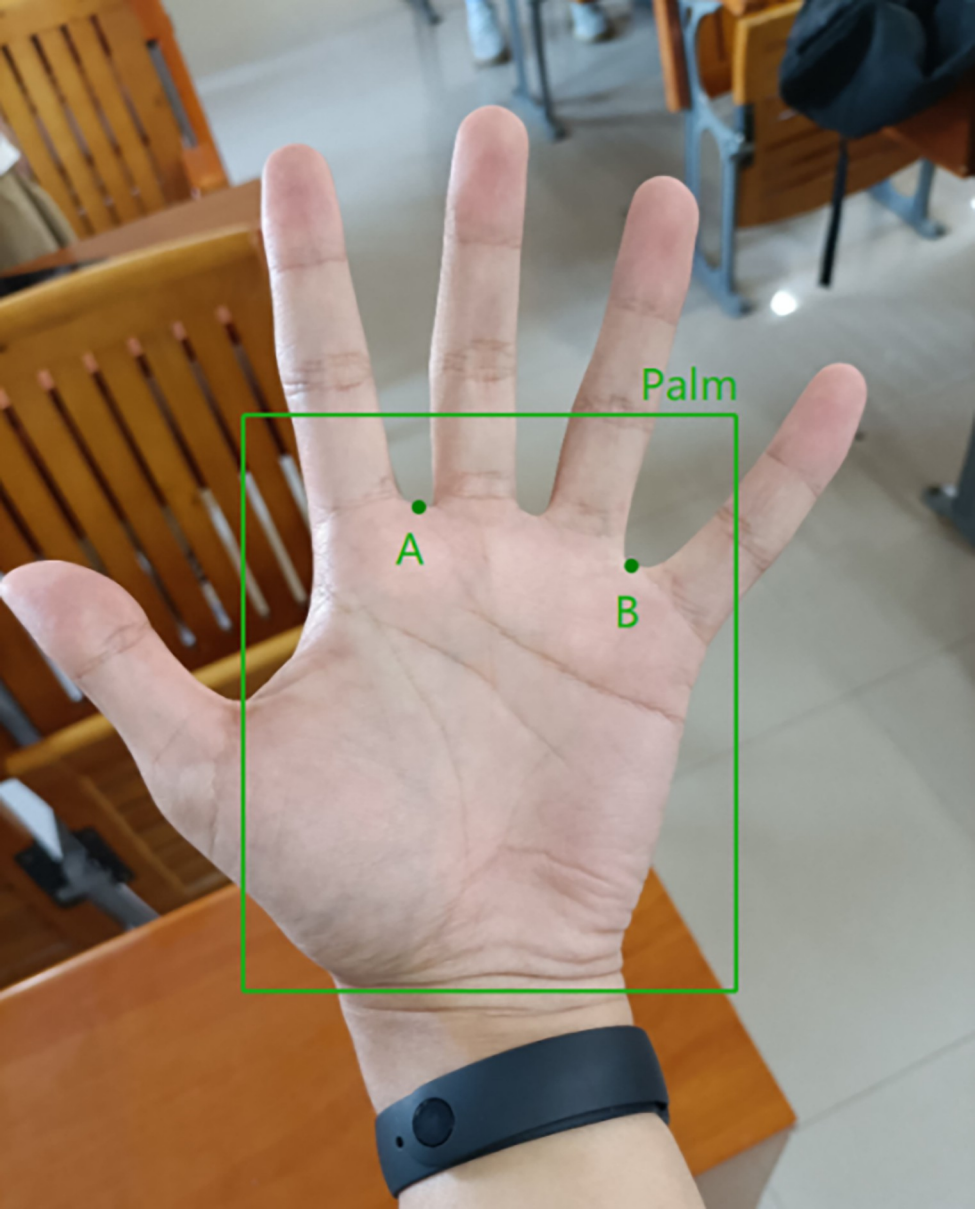
Example of data annotation.

Due to the use of two different networks in this article, it is necessary to label the data separately for both networks. As shown in Fig 14, for the object detection network, the annotation method is to mark a box at the position containing the palm, the first segment of the four fingers, and the valley of the fingers, representing "palm"; For the keypoint detection network, only the index finger middle finger valley point and the ring finger little thumb valley point are labeled and named point A and point B, respectively. In order to address the difficulty of ROI localization caused by different lighting, poses, and backgrounds in complex backgrounds, and to improve the practicality, robustness, and generalization ability of the model, the experimental dataset used was a mixture of five datasets mentioned in 2.2. Five datasets were randomly divided into training and testing sets in a 1:1 ratio, and then fused together to obtain the final mixed dataset. This is because training and testing on a single dataset often only reflects the performance of the model on that dataset, and a limited amount of data can lead to insufficient learning of features. When testing with other data, the results are often not good. In practical applications, there are often no images that are similar to the training and testing sets, so it is necessary to adopt a mixed training and testing method. The mixed database statistical table consisting of 5 databases is shown in Table 1. In addition, the dataset was expanded by rotating the images from 10° to 180° and 270°, and transforming the corresponding keypoint coordinates. The final result was a dataset with 19 times the number of samples compared to the original dataset. At the same time, during training, the algorithms library was used to randomly enhance the data, and operations such as randomly adjusting brightness, contrast, hue, saturation, randomly inverting image pixel values, and shifting up the RGB channel were performed to prevent overfitting and help the model learn more comprehensive features.

**Table 1:** Statistics of the dataset used in the experiment.

Database	Population	Size	Images	Training set	Test set
SCAUPD	89 university students	3000×4000	2274	1137	1137
REST	179 people aged 6–70	2048×1536	1948	974	974
MPD	200 people	3120×4160	16000	8000	8000
IITD	230 people aged 14–56	1600×1200	2600	1300	1300
BMPD	41 women	3264×2448	1640	820	820
Mixed Database	739people		24462	12231	12231

### 3.2 Palm initial localization experiment

#### 3.2.1 Evaluation metrics

In object detection networks, *IOU* is a metric used to measure the degree of overlap between the model detection box and the real target box, as shown in (13), where *A* and *B* represent the model detection box and the real target box, respectively. The *IOU* value range is between 0 and 1, indicating the degree of overlap between the predicted box and the actual target box.

As shown in (14), when the *IOU* is higher than a set threshold, it indicates accurate prediction, *P*(*x*) = 1, otherwise, the prediction is incorrect, *P*(*x*) = 0. The final formula for calculating the average accuracy is shown in (15), *AP*_*threshold* is the average accuracy under the set *threshold*, *n* is the total number of outputs of the model. The *threshold* is typically chosen between 0.5 and 0.95, based on practical considerations.


$$IOU=\frac{A\cap B}{A\cup B}$$ (13)

$$P(x)=\left\{ \begin{array}{c} 1\;IOU\geq threshold\\ 0\;IOU<threshold \end{array}\right.$$ (14)

$$AP\_threshold=\frac{\sum_{i=1}^{n}P(x)}{n}$$ (15)

#### 3.2.2 model training

**Fig 15:**
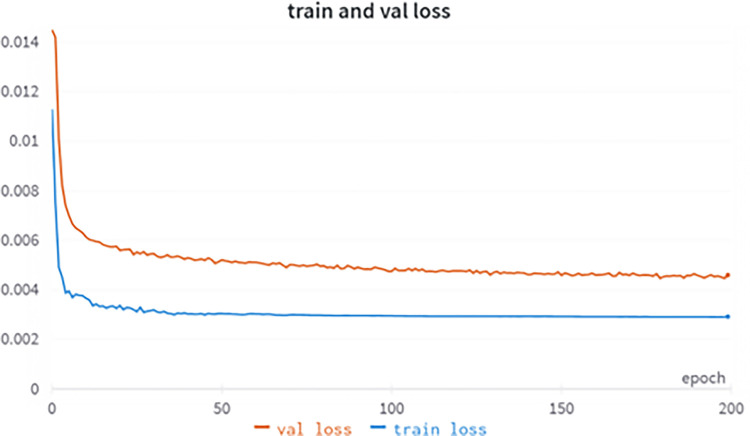
Training and validation loss curves for YOLOv5-lite.

**Fig 16:**
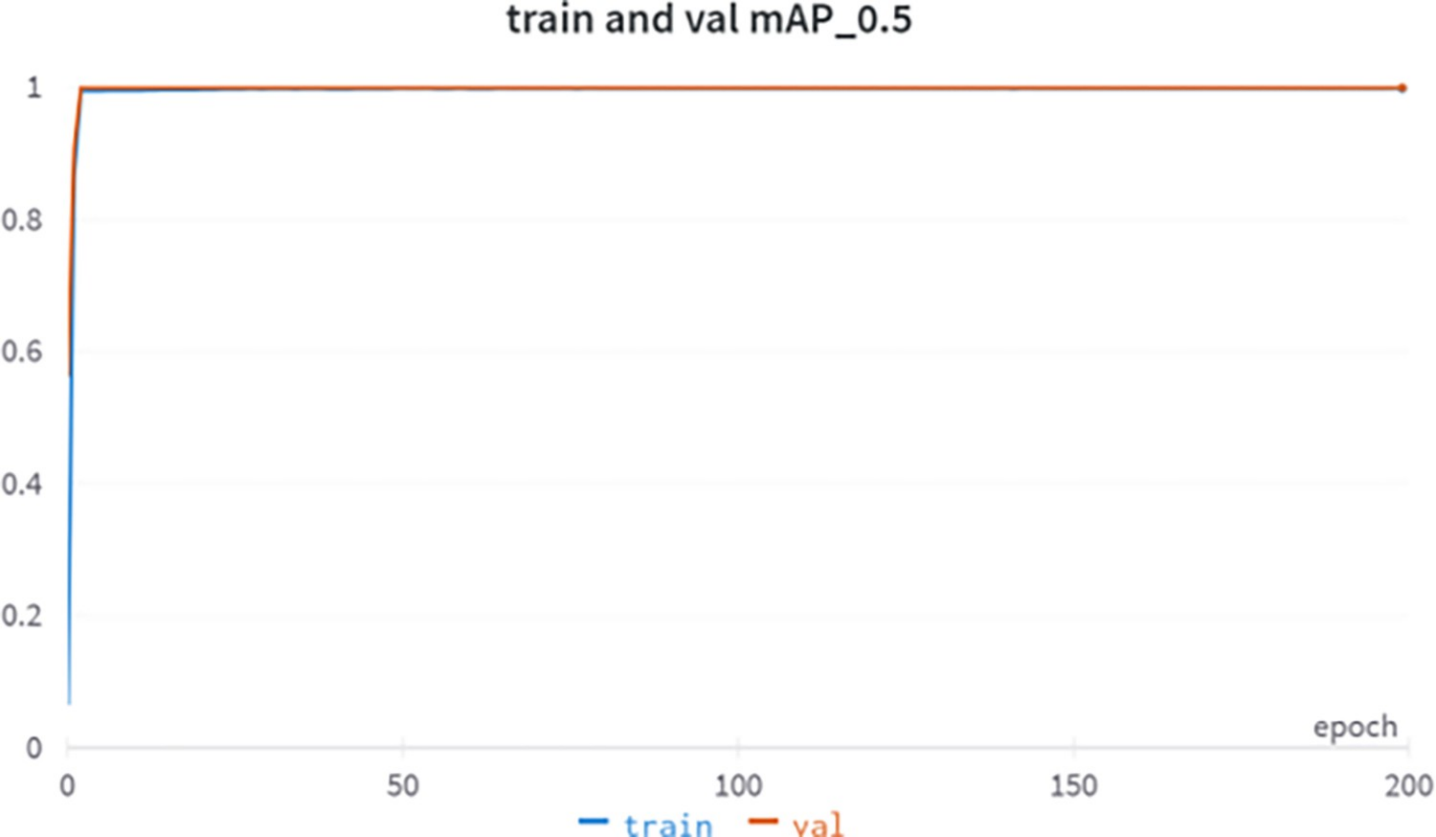
Training and validation accuracy curves for YOLOv5-lite.

The experiment was conducted using a Windows 10 64-bit operating system with an Intel(R) Core i7-10700F CPU with 7 GB of RAM, and an NVIDIA Quadro RTX 5000 graphics card using the Python and PyTorch frameworks. The training source code for YOLOv5-lite can be found on Github: https://github.com/ppogg/YOLOv5-Lite, where initial lr = 0.001, momentum = 0.937, weight decay = 0.0005, epoch = 200, batch size = 32, loss function for training and validation, mAP_ 0.5 is shown in Figs 15 and 16, respectively.

#### 3.2.3 Palm detection experiment

Compare the trained YOLOv5-lite with the YOLOv3-tiny used by previous studies [24] and [25] for palm recognition, as well as advanced lightweight detection networks such as YOLOv8-n [46], YOLOv9-t [47], and RT-DETR [48]. The experimental results are shown in Table 2. Since the goal of the object detection network in this stage is to detect only hands in the dataset without detecting other objects, the obtained mAP values are close to 100% and there is little difference among them, so they do not have high reference significance. Although the performance of each network is very similar, YOLOv5-lite stands out due to its extremely small scale and parameter count. Due to the fact that this stage only requires the simple task of detecting the palm, excessive parameter count and scale are unnecessary.

**Table 2:** Comparison of experimental results between YOLOv5-lite and other lightweight detection networks.

Method	mAP_0.5	Recall	Flops	Mean Time To Detection	Model Size	Parameters
YOLOv5-lite	99.91%	**100%**	**0.73G**	10ms	**1.6M**	**0.78M**
YOLOv3-tiny	99.89%	93%	6.96G	**4ms**	33M	6.06M
YOLOv8-n	99.94%	**100%**	8.19G	10ms	6.1M	3.01M
YOLOv9-t	**99.95%**	**100%**	7.85G	23ms	4.5M	2.01M
RT-DETR	99.91%	**100%**	107.99G	25ms	64.6M	32.81M

Since the current object detection algorithms are already quite mature, and this paper only uses them to complete a simple palm detection task, using YOLOv5-lite has achieved good results. Therefore, this part of the experimental work is not the focus of this paper.

### 3.3 Keypoints detection and ROI localization experiment

#### 3.3.1 Evaluation metrics

In object detection, accuracy is determined by calculating whether the IOU is higher than a threshold. However, in keypoints detection, accuracy is determined by calculating whether the Euclidean distance between the predicted keypoints coordinates and the ground truth labels is lower than an evaluation threshold. The evaluation metrics for the keypoints detection model are shown in (16) to (18). Among them, (*x*_*dt*_, *y*_*dt*_) is the predicted coordinates, (*x*_*gt*_, *y*_*gt*_) is the true label coordinates, *d* represents the Euclidean distance between the predicted coordinates of the model and the true coordinates, and *threshold* is the evaluation threshold. When the distance *d* between the two is less than or equal to the *threshold*, it indicates that the prediction is accurate, with *P*(*x*) = 1. Otherwise, it is considered a prediction failure, with *P*(*x*) = 0. After accumulating the accuracy of *n* outputs, the final average accuracy *Accuracy* is calculated.


$$d=\sqrt{{\left(x_{dt}-x_{gt}\right)}^{2}+{\left(y_{dt}-y_{gt}\right)}^{2}}$$ (16)

$$P(x)=\left\{ \begin{array}{c} 1\;d\leq threshold\\ 0\;d>threshold \end{array}\right.$$ (17)

$$Accuracy=\frac{\sum_{i=1}^{n}P(x)}{n}$$ (18)

#### 3.3.2 Model training

**Fig 17:**
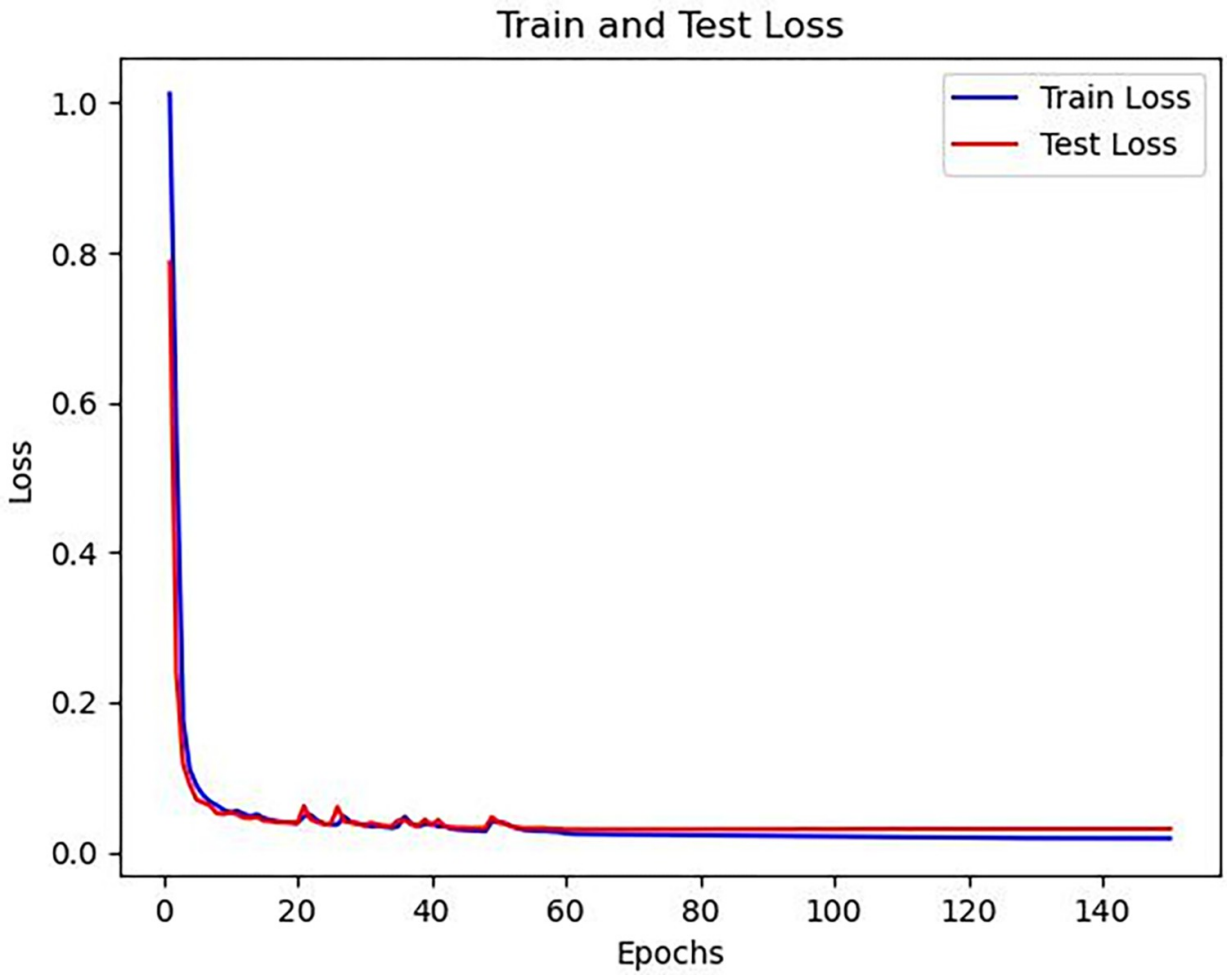
Training and testing loss function curves for the improved UNet model.

**Fig 18:**
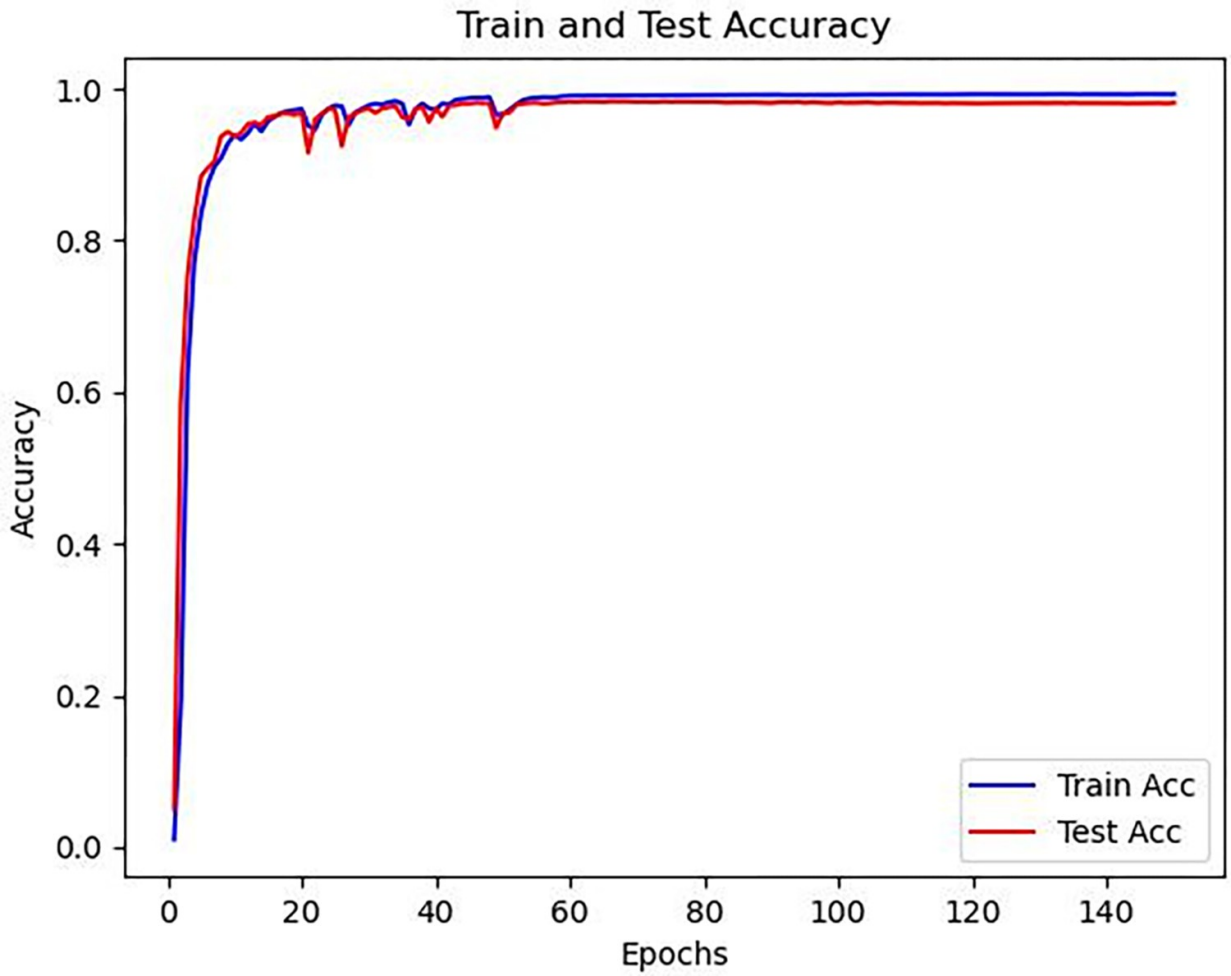
Training and testing accuracy curves for the improved UNet model.

The experimental environment and database used in this study are the same as those in Section 3.2. The training was performed for 150 epochs with a batch size of 512. The initial learning rate was set to 0.001, and an adaptive learning rate method was employed. The learning rate was automatically reduced to a half of its original value every 30 epochs. The accuracy evaluation metrics were set as mentioned in the previous section, with values of 1, 1.5, and 2.When using a value of 1.5, the training and validation loss functions and accuracy are shown in Figs 17 and 18, respectively. The final training accuracy reached 99%, while the test accuracy reached 98.3%.

#### 3.3.3 Keypoints detection and ROI localization experiment

**Fig 19:**
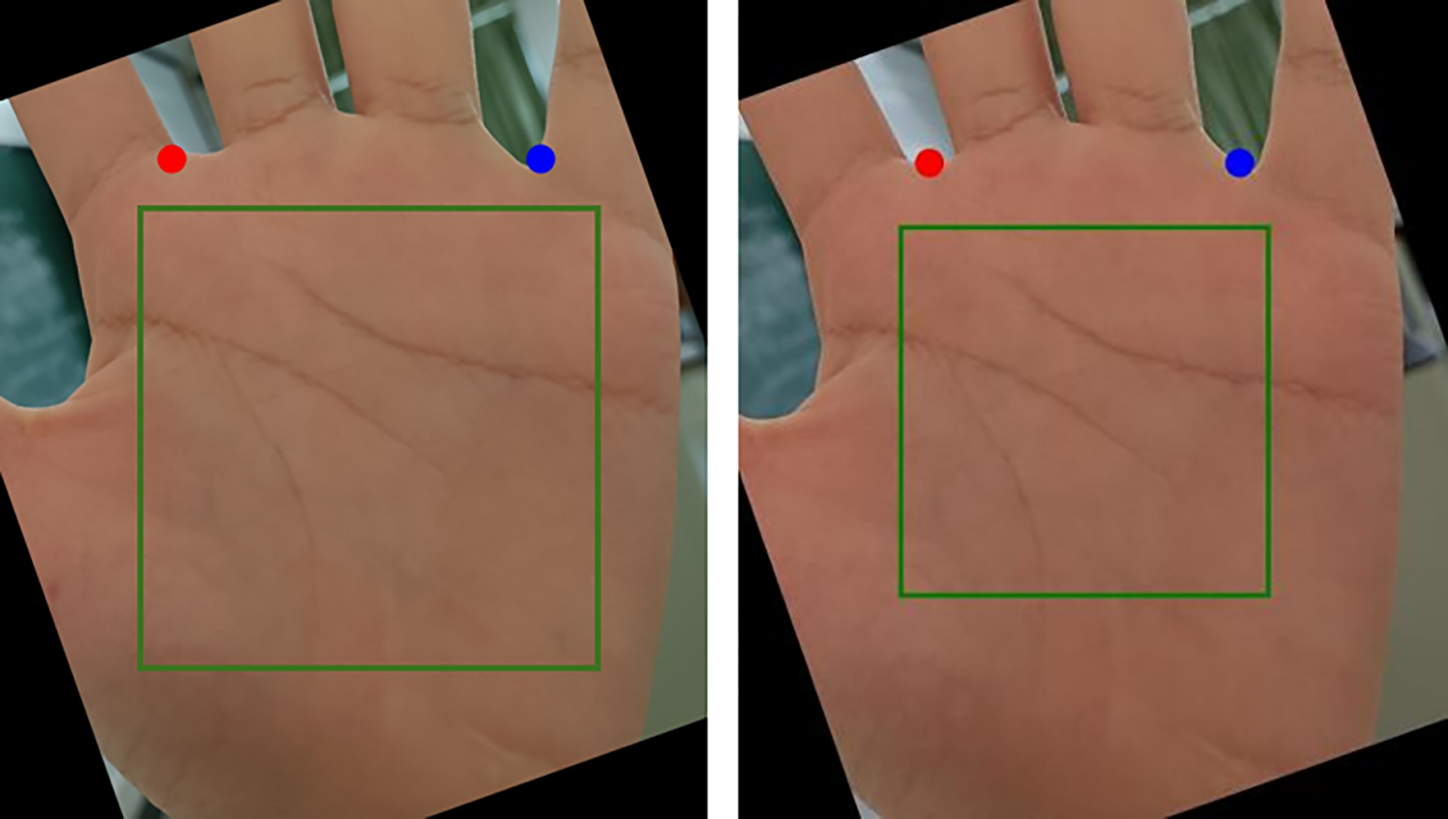
Threshold = 2 model output image (left) and normal label model output image (right).

From (12), it can be inferred that for the same model, different values of the threshold will affect the final detection accuracy. Therefore, experiments were conducted with threshold values of 1, 1.5, and 2. The experimental results are shown in Table 3. The test accuracies were 93.1%, 98.3%, and 99% for threshold values of 1, 1.5, and 2, respectively. Since the feature map size of the model is 64x64, a threshold of 2 would be too large and result in biased accuracy. On the other hand, a threshold of 1 would be too strict and classify many acceptable error points as incorrect. Therefore, in this study, a threshold of 1.5 was chosen. When threshold = 2, it is possible to output key points as shown in Fig 19. The two points deviate far from the valley point, resulting in a large ROI area being located. When performing multiple ROI localization with the same hand, if the difference in ROI area captured each time is too large, it may affect subsequent feature extraction. In addition, when using a larger distance rule (such as ROI edge length = 1.5|AB|), it is possible to cause the ROI region to detach from the palm, containing irrelevant information for subsequent feature extraction.

**Table 3:** Model training accuracy and test accuracy under different thresholds.

Threshold	Training Accuracy	Test Accuracy
1	99.3%	93.1%
1.5	99.3%	98.3%
2	99.8%	99%

**Fig 20:**
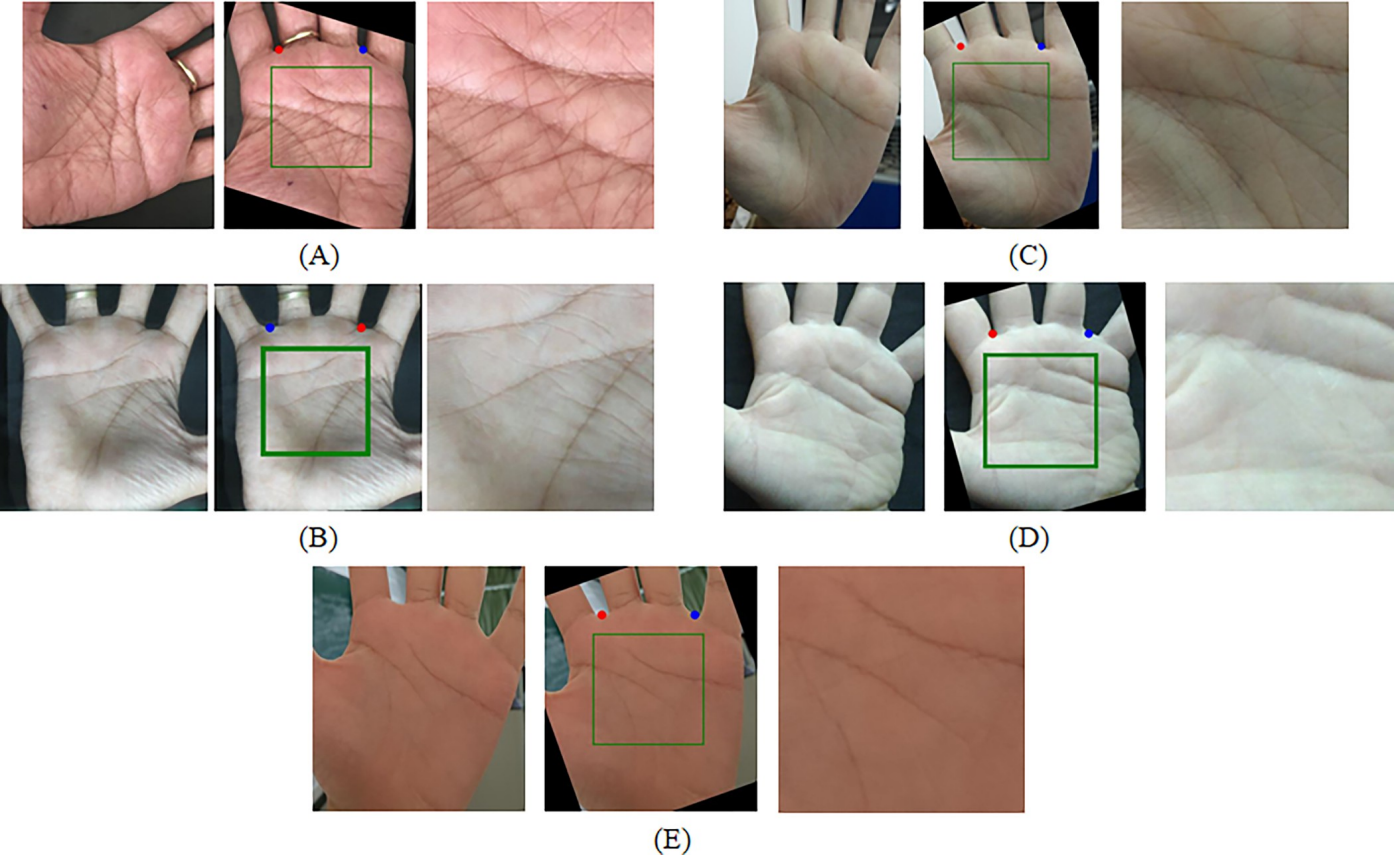
The implementation effect of the model on different datasets when Threshold = 1.5. (A) is BMPD, (B) is IITD, (C) is MPD, (D) is REST, and (E) is SCAUPD.

The detected keypoints on various datasets are shown in Fig 20. The red keypoint represent point A, which is the valley point between the index and middle fingers. The blue keypoint represent point B, which is the valley point between the ring and little fingers.

### 3.4 Ablation experiment

#### 3.4.1 Loss function ablation experiment

**Fig 21:**
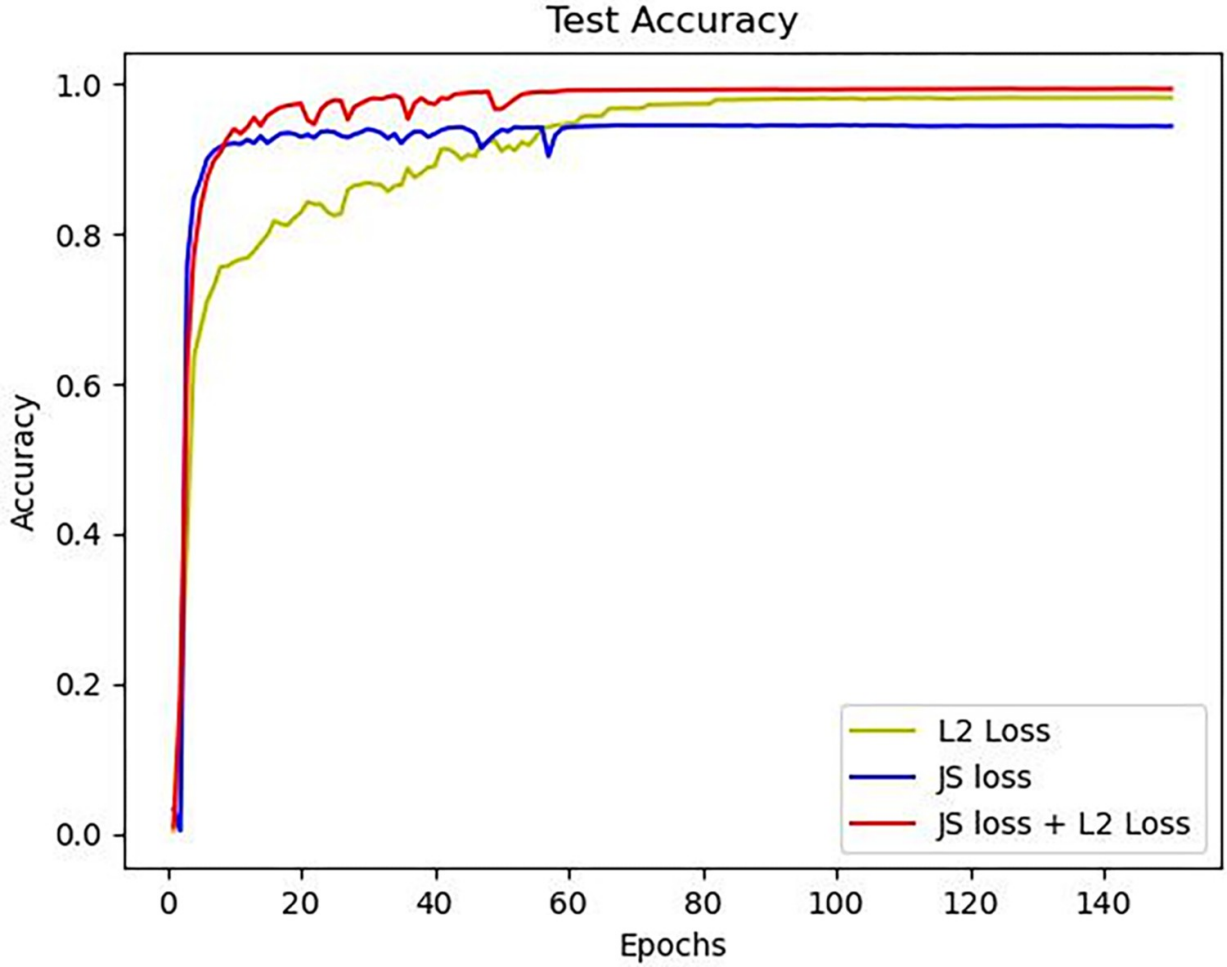
Convergence curves of model accuracy under different loss functions.

Table 4 records the accuracies obtained using three different approaches: direct regression based on L2 loss, heatmap regression based on JS loss, and a combination of both. Fig 21 compares the test accuracy variations for these three different loss functions. It can be observed that direct regression based on L2 loss has slower convergence, while heatmap regression based on JS loss achieves faster convergence. However, the final test accuracy of the JS loss approach is lower compared to the L2 loss approach. The combined approach achieves higher accuracy and is also relatively easier to train.

**Table 4:** Comparison of test accuracies for the model under different loss functions.

L2 Loss	JS Loss	Accuracy
√		97.5%
	√	94.3%
√	√	98.3%

#### 3.4.2 Module ablation experiment

**Fig 22:**
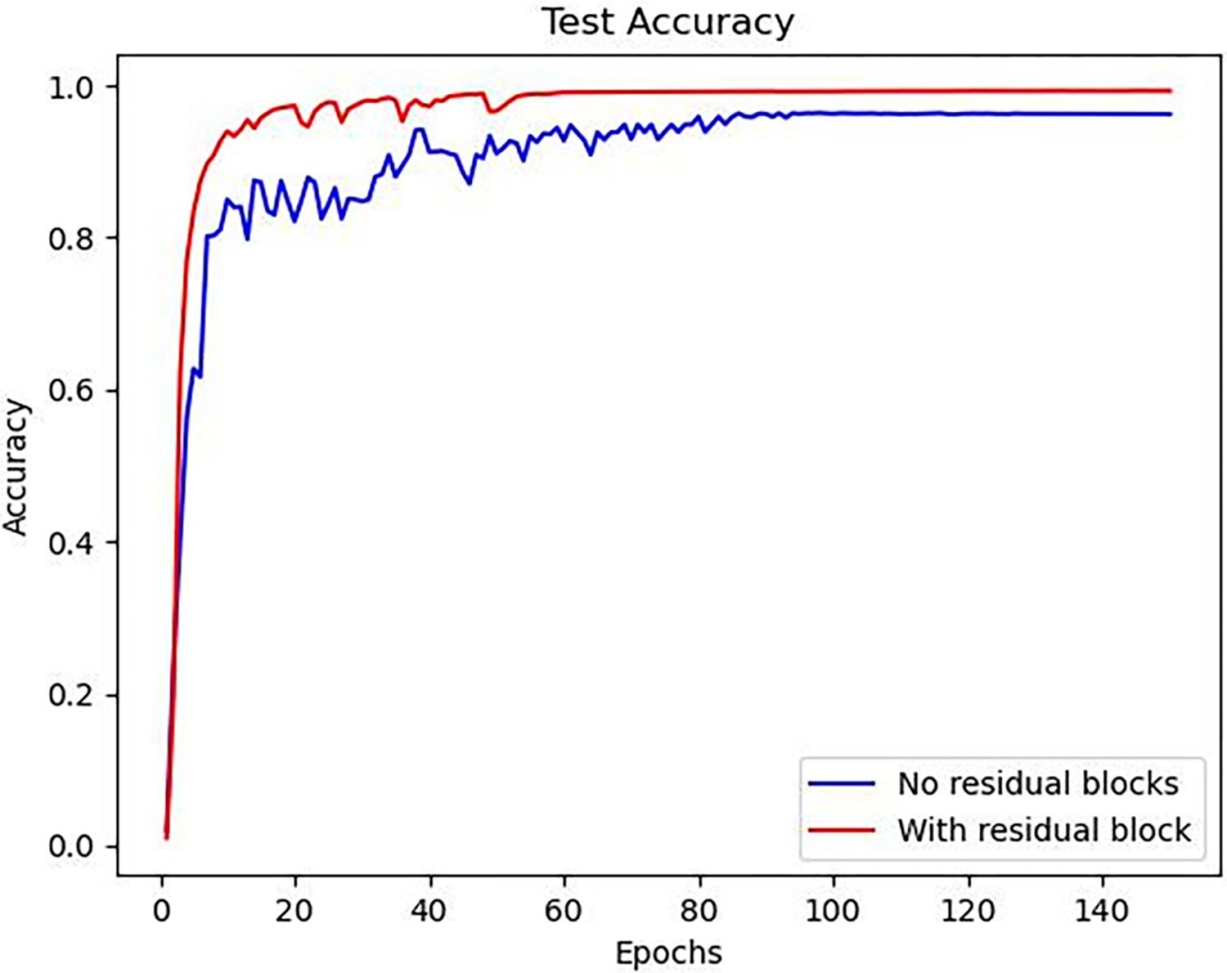
Convergence curve of accuracy for the residual block ablation experiment.

After adopting depth-wise separable convolutions, although the accuracy decreased from 98.5% to 98.3%, the model size reduced significantly from 1.22M to 871k, which is a reduction of approximately one-third. The decrease in accuracy was only 0.2%, which is within an acceptable range. This achieved a lightweight improvement in the model. When downsampling using the conventional convolutional and max-pooling method employed by the original UNet, the accuracy curve during testing is shown in Fig 22, and the detection results are presented in Table 5. The results indicate that the network without residual downsampling not only has slower convergence speed during training compared to the network with residual downsampling but also has lower final accuracy. This confirms the earlier claim that combining residuals with downsampling can address network degradation issues and accelerate convergence. Analyzing the average detection time of the network reveals that although using residual blocks complicates the network and increases the average detection time, the use of depth-wise separable convolutions effectively speeds up network computations, compensating for this issue.

**Table 5:** Comparison of model size, average detection time, and testing accuracy using different modules.

Residual Downsampling	Depthwise Separable Convolution	Model Size	Average Detection Time(GPU)	Accuracy
		1.17M	28ms	94.9%
√		1.22M	29ms	98.5%
	√	783k	27ms	94.6%
√	√	831k	28ms	98.3%

### 3.5 Comparison with other methods

#### 3.5.1 Comparison with other deep learning networks

**Fig 23:**
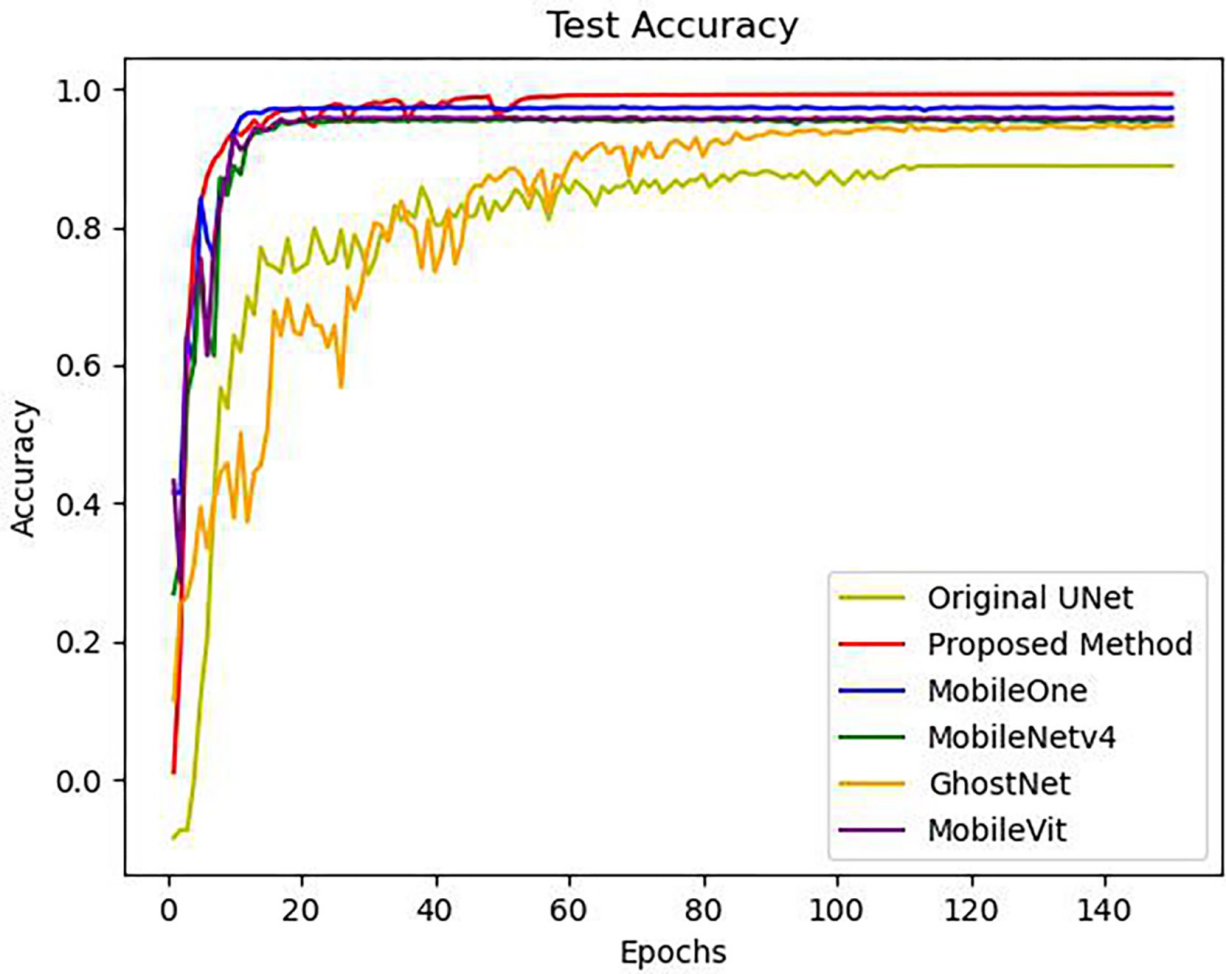
Diagram of changes in testing accuracy between the proposed network and other networks.

Compare the proposed keypoint detection network with the original UNet and mainstream and advanced lightweight networks such as GhostNet [49], MobileViT [50], MobileOne [51], MobileNetv4 [52]. The test results are shown in Table 6 and Fig 23. Among them, MobileViT uses the xxs version, MobileOne uses the small version, and MobileNetv4 uses the s_0_ version, all of which are the lightest versions among them. Please note that the networks used in this experiment were not pre trained. From Table 6, it can be seen that the proposed network outperforms other networks in terms of model size, average detection time, and accuracy. The original UNet adopted a method of gradually doubling the number of feature maps during the downsampling process with increasing layers. After 4 downsampling cycles, the number of feature channels increased from 64 to 1024, which undoubtedly added many redundant features. The original UNet accuracy was only 88.7%, which is due to the excessive number of feature channels and the difficulty of the model in learning truly effective information; At the same time, the network is too large, which greatly increases the detection time and is not conducive to real-time detection. As can be seen from Fig 23 the learning process of other lightweight networks is highly unstable, indicating that the model may have learned incorrect features. The convergence process of the proposed network is very stable, indicating that the model has indeed learned the correct features.

**Table 6:** Comparison between the proposed network and other networks.

Database	Keypoints detection network	Model size	Average detection time (GPU)	Accuracy
Mixed Database	Proposed method	**831k**	**28ms**	**98.3%**
	Original UNet	67.6M	64ms	88.7%
	GhostNet	15.5M	33ms	94.6%
	MobileOne	17.0M	29ms	97.3%
	MobileNetv4	9.72M	29ms	95.5%
	MobileViT	3.98M	34ms	95.9%

The reason why the proposed network is effective is that it is designed for specific key point detection tasks, while other mainstream lightweight networks are proposed to serve object detection or classification, and the effect is naturally not ideal. In addition, other lightweight networks use convolutional and fully connected layers for output. After multiple downsampling, the feature maps become very small, and finally, they are classified through fully connected layers. This is suitable for object detection or classification tasks, but not suitable for keypoints detection tasks. This is because the final output of the object detection task is the detection box of a certain target object, the classification task outputs the representative number of a certain category, and the keypoint detection task needs to output a point with spatial information, not a single pixel representing coordinates. Therefore, the encoder decoder network is more suitable for key point detection tasks, which also confirms the previous statement. Meanwhile, other lightweight networks have larger scales and deeper layers, resulting in longer average detection times.

#### 3.5.2 Comparison with other ROI extraction methods

To verify the superiority of the proposed method, we compared it with Xu [26]’s PKLNet, Izadapanahkakhk [21]’s IzadNet (abbreviated), and Li [53]’s BPFNet using the RPG1K dataset. RPG1K is a collection of 1000 real-world palm pose and background images by Xu [26]. The first half of the 500 images were captured in natural scenes using smartphones, while the last 500 images were collected from the internet. All images are normalized to 640×480 for unified testing. This test is an open set test, and the training sets of all four networks do not contain images of RPG1K. This is to simulate the generalization ability of the proposed network in real-world application scenarios.

The introduction of the testing indicators used is as follows:

Positioning Error (LE): The distance between the predicted keypoint coordinates and the GT coordinates.

Normalized LE (NLE): To avoid changes in image size between different datasets, the LE value is normalized by dividing by the corresponding ROI edge length.

Success rate (SR): The percentage of correctly processed samples. Here, "correct" means NLE is less than 20%.

The experimental results are shown in Table 7, where the proposed method has an SR of 93%, which is superior to other networks; Moreover, from the mean, standard deviation, and median values of the positioning error LE statistics, it can be seen that the key points of positioning are roughly located near the GT coordinates and will not deviate too far, indicating that the model can correctly distinguish valley point features from other features. The positioning error is very low compared to other methods and has superior performance. The reason why this method has such high accuracy and generalization ability is attributed to its unique two-stage detection method. By using YOLOv5-lite for initial positioning of the palm, the interference of most irrelevant features can be removed, enabling the second stage keypoint network to accurately locate keypoints. The key point detection network in the second stage combines speed and accuracy, and can quickly capture the features of finger valleys for accurate and efficient ROI extraction.

**Table 7:** Open set testing of the proposed method compared to other advanced methods.

Methods	SR(%)	LE(pixel)			Image length(pixel)
		Mean	Std	Median	
IzadNet	68.7	33.478	37.839	20.359	640
BPFNet	80.2	25.072	36.516	12.763	640
PKLNet	82.1	22.289	37.825	7.734	640
Proposed method	**93.4**	**5.52**	**12.1**	**3.325**	640

**Fig 24:**
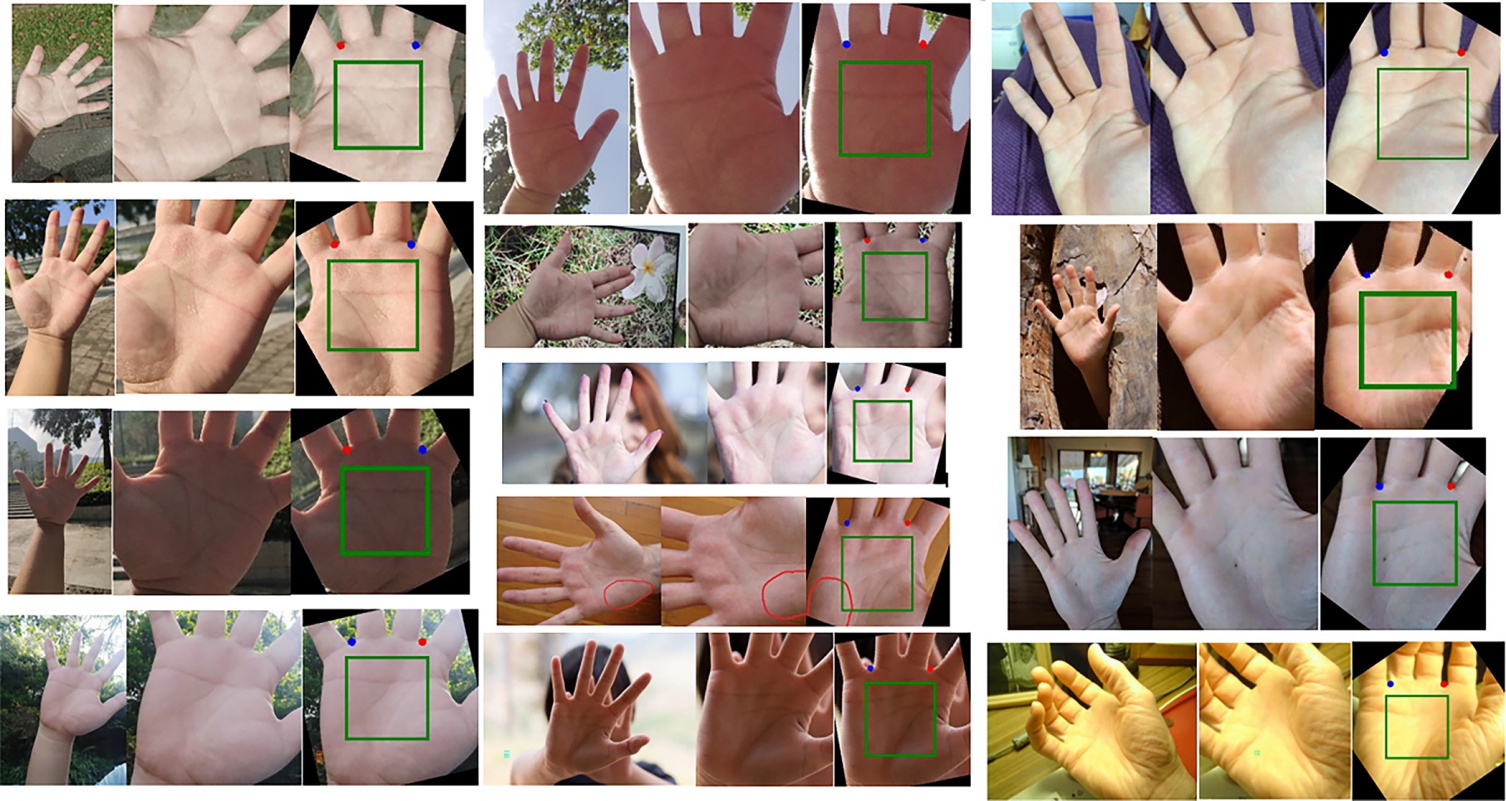
The performance of the proposed method on the RPG1K dataset.

The ROI instance diagram of the proposed method on RPG1K is shown in Fig 24. It can be seen that after the initial palm location in the first stage, complex features can be cleverly transformed into features similar to those in the training set. So, whether it’s pictures taken in real life or some unique images on the internet, they can be accurately recognized. Therefore, the proposed method has strong robustness and generalization ability.

**Fig 25:**
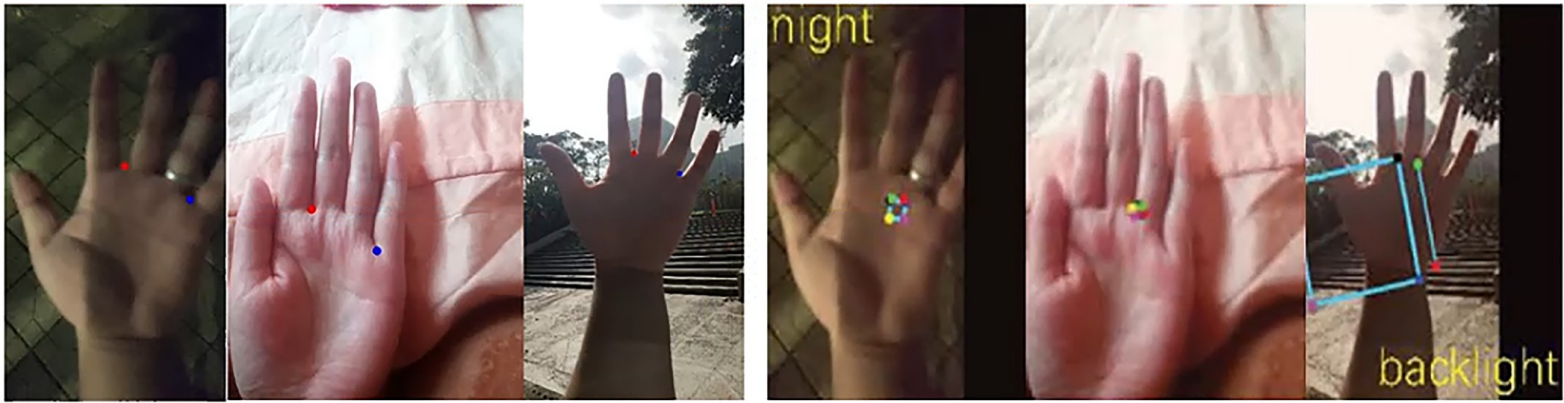
The performance of the proposed method in the example of PKLNet [26] localization failure.

Fig 25 shows some examples of localization failures in PKLNet (Fig 25 right, sourced from [26]) and the performance of the proposed method on those images (Fig 25 left). It can be seen that the proposed method can accurately detect keypoints and extract ROI regions in special situations such as finger closure, backlighting, and nighttime. For the convenience of calculating LE and comparing effects, we display keypoints from the original image through coordinate transformation.

The scale and average detection time of each model are shown in Table 8. Among them, the model size of the proposed method is the size of YOLOv5-lite plus the improved UNet size, which is 2.3MB. Due to the image size of 640 in the RPG1K dataset × 480 is much smaller than the average image size of the mixed dataset, so the average detection time of the proposed method is much faster than that on the mixed database, with 5.95ms (GPU) and 15.97ms (CPU), respectively. It is better than PKLNet and BPFNet, slightly slower than IzadNet’s 1.08ms on GPU, but faster than its 27.35ms on CPU. And the model size is only 2.3MB, which is much smaller than the above three networks, and can perform real-time, fast, and accurate ROI localization.

**Table 8:** The average detection time and model size of the proposed method compared to other advanced methods.

Methods	Average detection time (GPU)	Average detection time (CPU)	Model size (MB)
IzadNet	**1.08ms**	27.35ms	228.10MB
BPFNet	13.06ms	167.52ms	105.76MB
PKLNet	29.17ms	245.58ms	66.91MB
Proposed method	5.95ms	**15.97ms**	**2.4MB**

## 4 Conclusion

This article proposes a method for extracting the region of interest (ROI) in unconstrained palmprints based on lightweight neural networks. It aims to address the challenges of extracting the ROI in palmprints affected by factors such as hand pose and angle, complex background, and lighting conditions in unconstrained environments. To achieve real-time and fast palmprint recognition on embedded devices, the proposed method employs lightweight neural network models. Inspired by pedestrian detection in face recognition, this article introduces a two-stage method for extracting the ROI in palmprints. First, it detects whether there is a palm in the target image, and then it performs keypoints detection on the detected palm to extract the ROI. This two-stage approach filters out most irrelevant interferences in the background during palm detection in the first stage, facilitating subsequent ROI extraction. It also saves computational resources by stopping the recognition process when there is no palm in the target image. The keypoints detection network is based on the encoder-decoder structure of UNet and introduces residual blocks during the downsampling process to mitigate degradation issues caused by network deepening. To achieve lightweight design, the original UNet’s design of exponentially increasing feature channels during downsampling is abandoned in favor of a fixed number of feature channels. Additionally, depth-wise separable convolutions are used to replace most of the convolutions. Unlike traditional keypoints detection methods, the proposed method combines Gaussian heatmap regression and direct coordinate regression. Gaussian heatmap regression classifies the region containing keypoints instead of individual pixels, which helps the network better learn the ridge features and accelerates model convergence. Direct coordinate regression compensates for the lack of precision in Gaussian heatmap regression, leading to higher recognition accuracy. Therefore, a joint loss function is proposed, combining JS loss and L2 loss for training. The method achieves a keypoints recognition accuracy of 98.3% on a mixed dataset combining five datasets, with an average detection time of only 28ms. Experiments have shown that the keypoint localization model outperforms existing lightweight models in terms of speed, accuracy, and model size. In open set testing, compared with other state-of-the-art ROI extraction methods, the proposed method has the highest success rate, the lowest localization error, and the smallest model size. The proposed method has a success rate of 93.4%, an average detection time of 5.95ms on the GPU, and a model size of 2.4MB, far ahead of the latest palm print ROI extraction algorithm PKLNet’s 82.1% success rate, an average detection time of 29.17ms on the GPU, and a model size of 66.9MB. It can achieve fast, accurate, and lightweight palm print ROI extraction, and has strong robustness and generalization ability. The follow-up research of this study will continue to improve YOLOv5-lite and improved UNet to achieve smaller scale and higher accuracy, while deploying applications in embedded devices to achieve true application implementation.
